# Designing Ductile 2‐GPa Yielding Titanium Alloys via Multifunctional Subgrain Boundaries and Nanoprecipitates

**DOI:** 10.1002/advs.202519918

**Published:** 2025-11-30

**Authors:** Dingxuan Zhao, Kai Zu, Xu Yue, Hang Zhang, Zexuan Li, Keer Li, Zehua Zheng, Jialuo Yang, Wei Chen, Jinyu Zhang, Jun Sun

**Affiliations:** ^1^ State Key Laboratory for Mechanical Behavior of Materials Xi'an Jiaotong University Xi'an 710049 P. R. China; ^2^ Xinjiang Xiangrun New Materials Technology Co., Ltd. Hami 839000 P. R. China

**Keywords:** ductility, precipitate, strength, subgrains, titanium alloys

## Abstract

Ductile titanium alloys yielding at 2 GPa are rarely achieved via strengthening of hexagonal close‐packed *α*‐nanoprecipitates, which suffer from the planar glide softening to cause strain localization for insufficient work hardening capability. In this study, by engineering multifunctional *β*‐subgrain boundaries, an unprecedented combination of ultra‐high yield strength ≈1929 MPa and ultimate strength ≈2014 MPa, along with a notable uniform elongation ≈6.2%, is successfully achieved in a Ti‐4Al‐5Mo‐3V‐5Cr‐1Fe alloy. This superior mechanical performance is enabled by a unique microstructure featured with ultrafine *β*‐subgrains containing intragranular *α*‐nanoprecipitates and intergranular discrete *α*‐nanolaths. The resulting complex, multiscale hierarchical architecture effectively impedes and regulates dislocation motion, thereby endowing the alloys synergistic strengthening and ductilizing. Furthermore, local chemical heterogeneities combined with high stress‐driven elemental partitioning exert strong nanoscale segment detrapping effects on mobile dislocations, contributing to a sustained work hardening rate and thus large uniform elongation. This multifunctional subgrain boundary strategy holds significant promise for extension to other metallic materials, particularly these additively manufactured alloys with dense dislocation cells, toward achieving ultrastrong‐yet‐ductile performance.

## Graphical abstract

By engineering ultrafine *β*‐subgrains and nanoscale *α*‐precipitates in titanium alloys, the unique architecture achieves an unprecedented ultra‐high strength of 2 GPa while maintaining a notable uniform elongation of 6%, a combination that is both rare and highly promising for structural applications of titanium alloys. This microstructure design strategy holds significant potential for extension to other metallic materials, enabling superior strength‐ductility synergy.

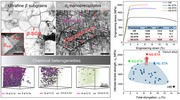

## 1 Introduction

Ductile alloys yielding at stresses (*σ_y_*) on the order of 2 GPa while retaining adequate uniform elongation (UE, *ε_u_*) ≥5% are highly desirable in structural applications. The state‐of‐the‐art alloys reached the yield strength (YS) *σ_y_* of 2 GPa are a few ultrastrong steels^[^ ^1^, ^2^, ^3^ ^]^ and multi‐principal element alloys,^[^ ^4^, ^5^, ^6^ ^]^ both of which have high mass density (*ρ*) (or low specific yield strength, SYS, *σ_y_*/*ρ*) or plastic instability (such as Lüders strains) rather than truly uniform elongation. In general, the Lüders strain is caused by the catastrophic release of abundant mobile dislocations, associated with low work hardening rates (WHRs, *θ*).^[^ ^1^, ^3^, ^5^ ^]^ As Lüders bands are absent, such as in some 2‐GPa maraging steels reinforced by nanoprecipitates, the *ε_u_* is limited to only ≈3.8%.^[^ ^2^ ^]^

In response to the particular emphasis of high SYS, lightweight titanium (Ti) alloys have garnered great interest for their mechanical property optimization.^[^ ^7^, ^8^ ^]^ The creation of densely dispersed hexagonal close packed (HCP) *α*‐nanoprecipitates in the body‐centered‐cubic (BCC) *β‐*matrix is the most effective strengthening pathway in Ti alloys.^[^ ^9^, ^10^, ^11^ ^]^ It renders high YS *σ_y_* of ≈1.75 GPa at the expense of ductility (*ε_f_* <5%), in particular UE *ε_u_* <3%,^[^ ^12^ ^]^ since the stress concentration at the semi‐coherent *α/β* interfaces causes strain localization or cracking for the strength‐ductility conflict.^[^ ^9^, ^13^, ^14^ ^]^ Several methods have been proposed in terms of architecting hierarchical microstructures to rejuvenate ductility *ε_f_* (even *ε_u_*),^[^ ^15^, ^16^, ^17^, ^18^, ^19^ ^]^ whereas there is no success in the design of YS *σ_y_* ≈2 GPa Ti alloys with UE *ε_u_* >5%. For example, the Ti‐1Al‐8V‐5Fe alloys with hierarchical microstructure of micron‐scale primary and nanoscale secondary *α*‐precipitates within the *β*‐matrix have *σ_y_* ≈1.65 GPa and ductility *ε_f_* <6%.^[^ ^17^ ^]^ Specifically, the continuous grain boundary *α*‐layer (*α*_GB_) severely degrade the ductility of high‐strength Ti alloys.^[^ ^10^, ^13^, ^14^ ^]^ Another example is that the additive manufactured commercial Beta‐C Ti alloys (with only nanoscale *α*‐precipitates), in which dense screw dislocations trigger the formation of twinned *α*‐nanoprecipitates, have the *σ_y_* ≈1.55 GPa and UE *ε_u_* ≈ 5.4%.^[^ ^10^ ^]^ It is thus a grand challenge to devise a novel microstructure to meet the above requirements for lightweight 2‐GPa Ti alloys.

Considering that the *α*‐precipitates and the *β‐*grains are quite difficult to further refine to realize the desired 2‐GPa yield strength via conventional thermomechanical processing, one naturally thinks of the subgrains (or dislocation cellular structures) to notably enhance mechanical properties of alloys, which are often formed during either plastic deformation or additive manufacturing.^[^ ^20^, ^21^, ^22^ ^]^ This unique structure functions not only as the dislocation obstacle for high WHRs, but also as the dislocation source to deconcentrate stresses, thus simultaneously enhance strength and ductility.^[^ ^20^, ^21^, ^23^, ^24^ ^]^ Also, *β*‐subgrain boundaries (SGBs) promote the *α*‐precipitation in the fashion of discrete laths along SGBs during aging, rather than a continuous *α*_GB_‐layer in conventional Ti alloys^[^ ^25^, ^26^ ^]^ to avoid localized deformation nearby SGBs. At the same time, extremely dense *α*‐nanoprecipitates in a high‐volume fraction are essential for such 2‐GPa YS Ti alloys.^[^ ^10^, ^11^ ^]^ An effective strategy is to utilize *ω*‐precursors, which are substantially formed via concurrent compositional and structural adjustment during low temperature aging processes, to substantially enhance the nucleation density of *α*‐nanoprecipitates in Ti alloys.^[^ ^11^, ^27^, ^28^, ^29^, ^30^ ^]^ Specifically, the *ω*‐mediated *α*‐precipitation promotes the formation of coherent twin boundaries (CTBs) between *α*‐nanoprecipitates to alleviate the strain incompatibility of semi‐coherent *α/β* interfaces for enhanced ductility.^[^ ^27^, ^31^, ^32^ ^]^ Hence, apart from strengthening and ductilizing effects, multifunctional SGBs may play important roles in *ω*‐assistant *α*‐precipitation, being a promising strategy to enhance the ductility of ultrastrong Ti alloys.

To design 2‐GPa Ti alloys with *ε_u_* >5%, we here architect a novel microstructure consisting of dense *α*‐nanoprecipitates (*α_s_*) uniformly dispersed in ultrafine *β*‐subgrains (*β_sub_*) of a new commercial Ti‐4Al‐5Mo‐3V‐5Cr‐1Fe (wt.%) *β*‐Ti alloy. The outstanding mechanical properties mainly stem from dense *α_s_*‐nanoprecipitates to restrict dislocation motion for pronounced precipitation strengthening and high density SGBs, as well as high stress‐driven elemental partitioning to enhance work hardening capability for ductilizing. Through this combined strength and ductility‐enhancing mechanisms, the present Ti‐4Al‐5Mo‐3V‐5Cr‐1Fe alloy achieves an unprecedented synergy of record‐high YS *σ_y_* ≈1929 MPa and notable UE *ε_u_* ≈6.2%.

## 2 Results

### 2.1 Microstructural Design

The raw material used in this study is a commercial Ti‐4Al‐5Mo‐3V‐5Cr‐1Fe metastable *β*‐Ti alloy in the form of hot‐rolled wires with a diameter of 7 mm. To engineer ultrafine *β_sub_*‐grains and *α_s_*‐nanoprecipitates in the microstructure, we designed a combined processing route involving hot rolling (HR) and heat treatment. Specifically, the final HR process was conducted in the low‐temperature (*α*+*β*) duplex region to retain a high density of dislocations, which serves as a critical prerequisite for the formation of ultrafine *β_sub_*‐grains. The resulting microstructures in the wires are displayed in Figure S1 (Supporting Information). A short‐term solution treatment via water cooling (WC) near the *β*‐transus temperature (T*_β_* ≈810 °C) was used for these HR wires to preserve abundant dislocations for refining *α_s_*‐nanoprecipitates during subsequent dual‐aging. This treatment promotes dislocation rearrangement to form *β_sub_*‐grains while suppresses subgrain growth or recrystallization. Densely metastable *ω*‐precursors formed in the low‐temperature pre‐aging stage can create a high density of extremely fine *α_s_*‐nanoprecipitates during the subsequent high‐temperature aging, followed by air cooling (AC).^[^ ^27^, ^28^ ^]^ This route, i.e., ‘800 °C/10 min/WC + 350 °C/2 h/AC + 450 °C/10 h/AC’ for our Ti alloy (referred to as NS‐STA hereinafter) and the corresponding microstructural evolution are schematically illustrated in Figure S2 (Supporting Information).

To highlight the significance of combining *α_s_*‐nanoprecipitates with ultrafine *β_sub_*‐grains in achieving superior strength‐ductile synergy, two additional microstructures were constructed for comparison. These include *α_s_*‐nanoprecipitates combined with conventional coarse *β*‐grains, as well as *α_s_*‐nanoprecipitates paired with large *β_sub_*‐grains. For the former, the solution temperature was raised above T*_β_* to fully recrystallize *β_sub_*‐grains into *β*‐grains (820 °C/10 min/WC + 350 °C/2 h/AC + 450 °C/10 h/AC, hereinafter referred to as AS‐STA). For the latter, the solution treatment was still conducted near T*_β_* but with an extended duration to promote *β_sub_*‐grain growth and more dislocation annihilation (800 °C/1 h/WC + 350 °C/2 h/AC + 450 °C/10 h/AC, hereinafter referred to as NL‐STA).

### 2.2 Initial Microstructural Features

**Figure 1:**
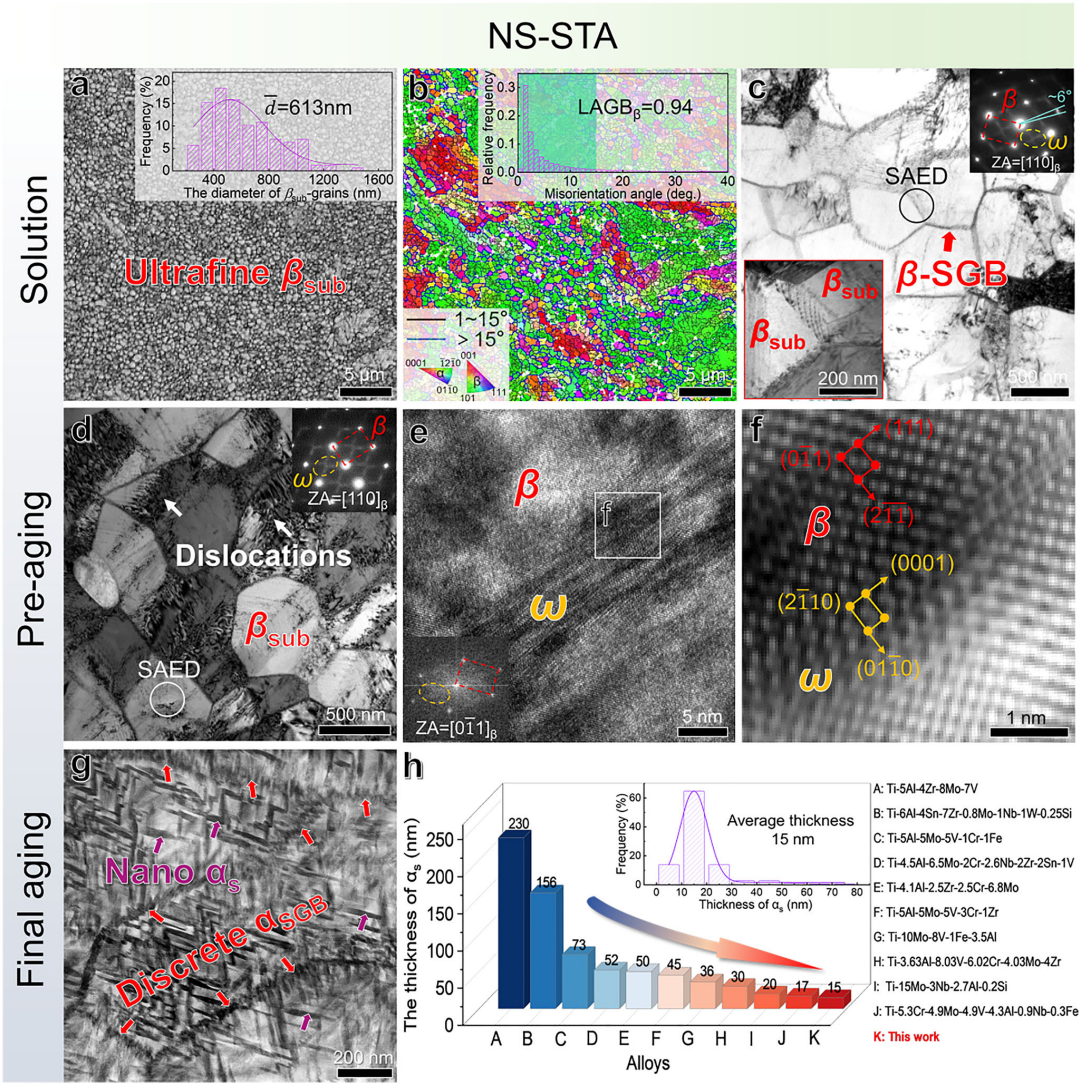
Morphological evolution of the NS‐STA samples subjected to sequential solid solution and dual‐step aging treatments. a–c) show the microstructures after near *β*‐transus solution treatment, while d–f) and g,h) show the microstructures following subsequent low‐temperature pre‐aging and high‐temperature final aging treatments, respectively. (a) the BC image and (b) the corresponding IPF map overlaid with an orientation map. The HAGB and the LAGB, marked in blue and black, correspond to misorientations greater or less than 15°, respectively. The insets in (a) and (b) present statistical histograms of *β_sub_*‐grain diameters and LAGB fractions, respectively. (c) the TEM BF‐image taken along the [110]*_β_* zone axis. The upper‐right inset presents the SAED pattern across two *β_sub_*‐grains indicated by the circle, while the lower‐left inset provides a high‐magnification view of *β*‐SGBs. (d) TEM BF‐image after the pre‐aging treatment. The inset is the SAED pattern of *β_sub_*‐grain marked by the circle. (e) HRTEM image of *ω*‐precipitates within *β_sub_*‐grains. The inset is corresponding FFT pattern. (f) IFFT image of *ω*‐precipitates taken from the frame in (e). (g) HAADF image after the final aging treatment. (h) A comparison of *α_s_*‐thickness for the current Ti alloy with other reported high‐strength Ti alloys.^[^ ^10^, ^11^, ^15^, ^16^, ^33^, ^34^, ^35^, ^36^, ^37^, ^38^ ^]^ The inset is a statistical histogram of the thickness distribution of our *α_s_*‐nanoprecipitates.

The NS‐STA samples develop their characteristic microstructures through a sequential process of specific solid solution and aging treatments. **Figure** **1**a shows the microstructure of the samples subjected to the short‐term solution treatment, consisting of numerous equiaxed *β_sub_*‐grains with well‐defined boundaries. The average diameters of *β_sub_*‐grains were measured to be 613 nm. Figure 1b reveals that low‐angle grain boundaries (LAGBs) account for ≈94% of all boundaries, implying that *β_sub_*‐grains are predominantly formed during the solution treatment. Figure 1c presents *β*‐SGBs consist of a regularly arranged array of individual dislocations. The selected‐area electron diffraction (SAED) pattern in Figure 1c shows slightly arc‐shaped spots with a spread angle of ≈6°, consistent with the LAGB characteristics of the *β_sub_*‐grains. Also, some retained dislocations were observed within *β_sub_*‐grain interiors, due to intentionally insufficient dislocation annihilation during short‐term solution treatment. A careful examination of the *β_sub_*‐grains shows the embryonic *ω*‐like structure,^[^ ^29^, ^30^ ^]^ verified by faint diffuse streaks in the SAED of Figure 1c, as well as Figure S3 (Supporting Information). After low‐temperature pre‐aging treatment, there is a concurrent compositional and structural adjustment within the embryonic *ω*‐like structure in *β_sub_*‐grains, as verified by the increased intensity of diffraction spots in Figure 1d. Figure 1e,f confirms that the isothermal *ω*‐phase forms, following the classical crystallographic orientation relationship with the *β*‐matrix.^[^ ^30^ ^]^

Figure 1g shows that the *β_sub_*‐grains remain clearly distinguishable after the final high‐temperature aging treatment, in which these *α_s_*‐nanoprecipitates exhibit chevron‐like pairs composed of two *α*‐variants. This configuration has been reported to originate from multiple‐site nucleation initiated by *ω*‐particles^[^ ^31^ ^]^ or pre‐existing dislocations within the microstructure.^[^ ^10^ ^]^ Each *α*‐variant in such pairs obeys the Burgers orientation relationship with the *β*‐matrix in Figure S4 (Supporting Information). Two *α_s_*‐variants in each pair maintain a twinning relationship, with twin boundaries (TBs) identified as $\;{\left\{10\bar{1}1\right\}}_{\alpha }$.^[^ ^10^ ^]^ This finding further confirms that the twinned *α_s_*‐nanoprecipitates are generated within the aged ultrafine *β_sub_*‐grains. At the *β*‐SGBs, *α_s_*‐precipitation is strongly correlated with individual dislocations from the special dislocation array (Figure 1g). The *α*‐phase preferentially nucleates and grows along dislocation lines in the fashion of nanoscale laths, forming discontinuous SGB‐*α* nanolaths (*α*_SGB_). These discrete *α*_SGB_ nanolaths have been identified as a collection of *α*‐variants with dissimilar orientations, which essentially arises from variant selection of the *α*‐phase caused by the unique dislocation configuration at *β*‐SGBs and its associated stress field.^[^ ^25^ ^]^ Within *β_sub_*‐grain interiors, a high density of *α_s_*‐precipitates forms, having the thickness ranging from 6 to 75 nm with an average value of 15 nm. This average *α_s_*‐thickness in the current Ti alloy is almost the smallest, compared with those reported ones in Figure 1h.

**Figure 2:**
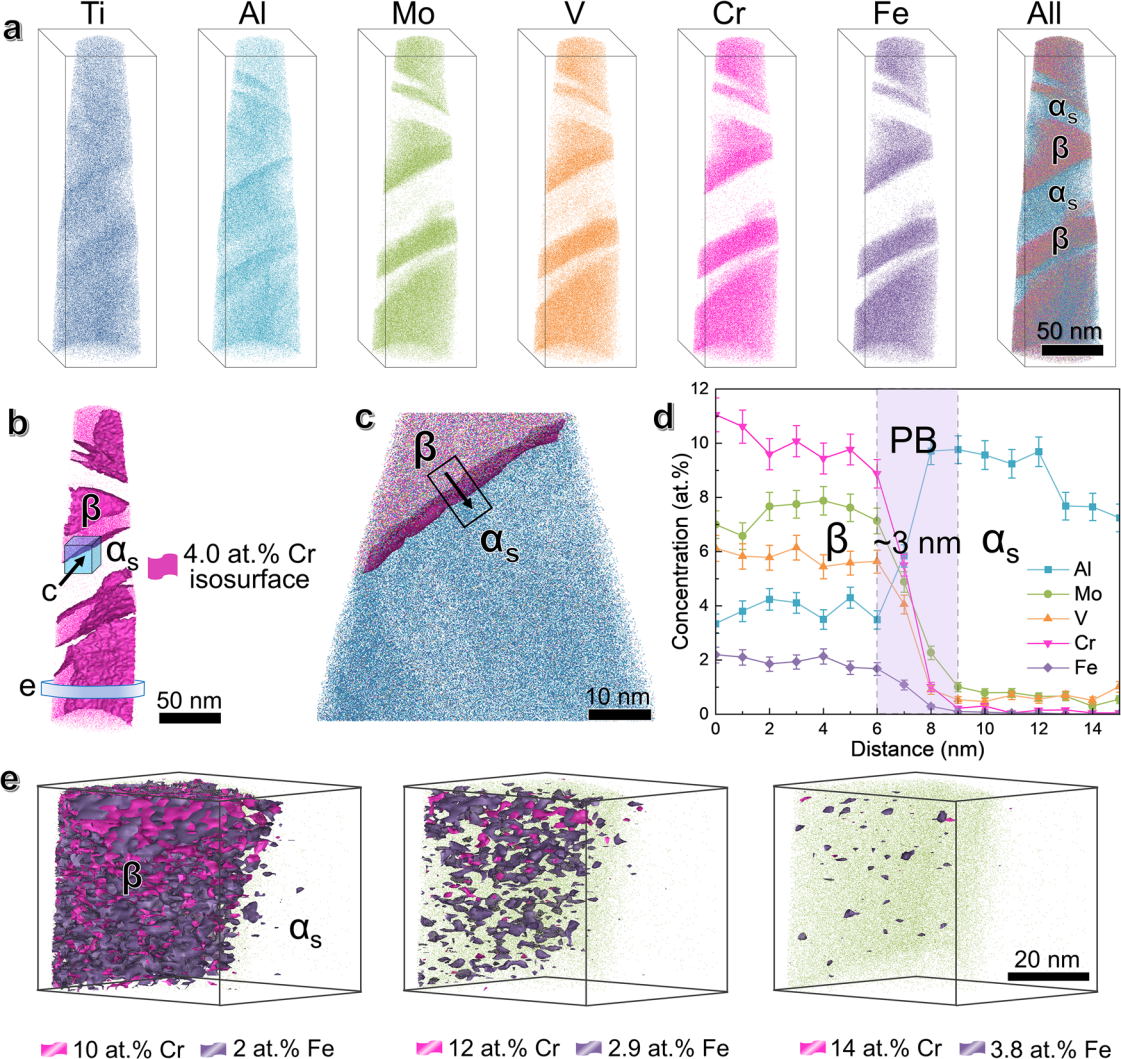
APT reconstruction and composition analysis of the NS‐STA samples prior to tension. a) The reconstructed APT atomic maps. b) 4.0 at.% Cr isoconcentration surface. c) An enlargement around PB between *α_s_*‐nanoprecipitate and the *β*‐matrix along the arrow in (b); d) Corresponding composition profile of Mo, V, Fe, Cr, and Al atoms measured across the arrow in c, showing the interfacial thickness of ≈3 nm. e) Iso‐concentration surfaces of the *β*‐matrix interior taken from the circle slice in (b), showing local chemical heterogeneities of nanoscale Cr and Fe enriched regions after defining various iso‐concentration surfaces.

The compositional distribution of both *α_s_* and *β* phases was analyzed using atom probe tomography (APT), see **Figure** **2**. The reconstructed atomic maps in Figure 2a show that the *β*‐stabilizers of Mo, V, Fe, and Cr elements are depleted from the *α*‐phase, all of which have the concentrations less than 1 at.%. In contrast, the *α*‐stabilizer Al exhibits a high concentration of 9 ± 1 at.% in the *α*‐phase and a low concentration of 4 ± 0.5 at.% in the *β*‐phase. The *α_s_*‐thickness is consistent with the TEM observations. Figure 2b, taking the most concentrated Cr element in the *β*‐matrix as an example, shows its iso‐concentration surface at 4.0 at.%. It appears the *α_s_*/*β* phase boundary (PB) is clear and sharp, with an average thickness of ≈3 ± 0.5 nm (Figure 2b,d). This PB feature is further verified by the 2D atomic distribution maps in Figure S5 (Supporting Information). Different iso‐concentration surfaces of Cr and Fe within the *β*‐grains, as shown in Figure 2e, reveal nanoscale compositional heterogeneities. This feature is further confirmed by the reconstructed atom maps in Figure S6 (Supporting Information), which exhibit slight nanoscale local chemical heterogeneities throughout the microstructure. These chemical heterogeneities in Fe and Cr are attributed to their thermodynamically favorable positive mixing enthalpies with Ti, the relatively high binding energies of Fe─Fe and Cr─Cr compared to Ti─Ti, as well as their intrinsic nature as *β*‐eutectoid forming elements. For further details, please refer to Section S1 (Supporting Information).

**Figure 3:**
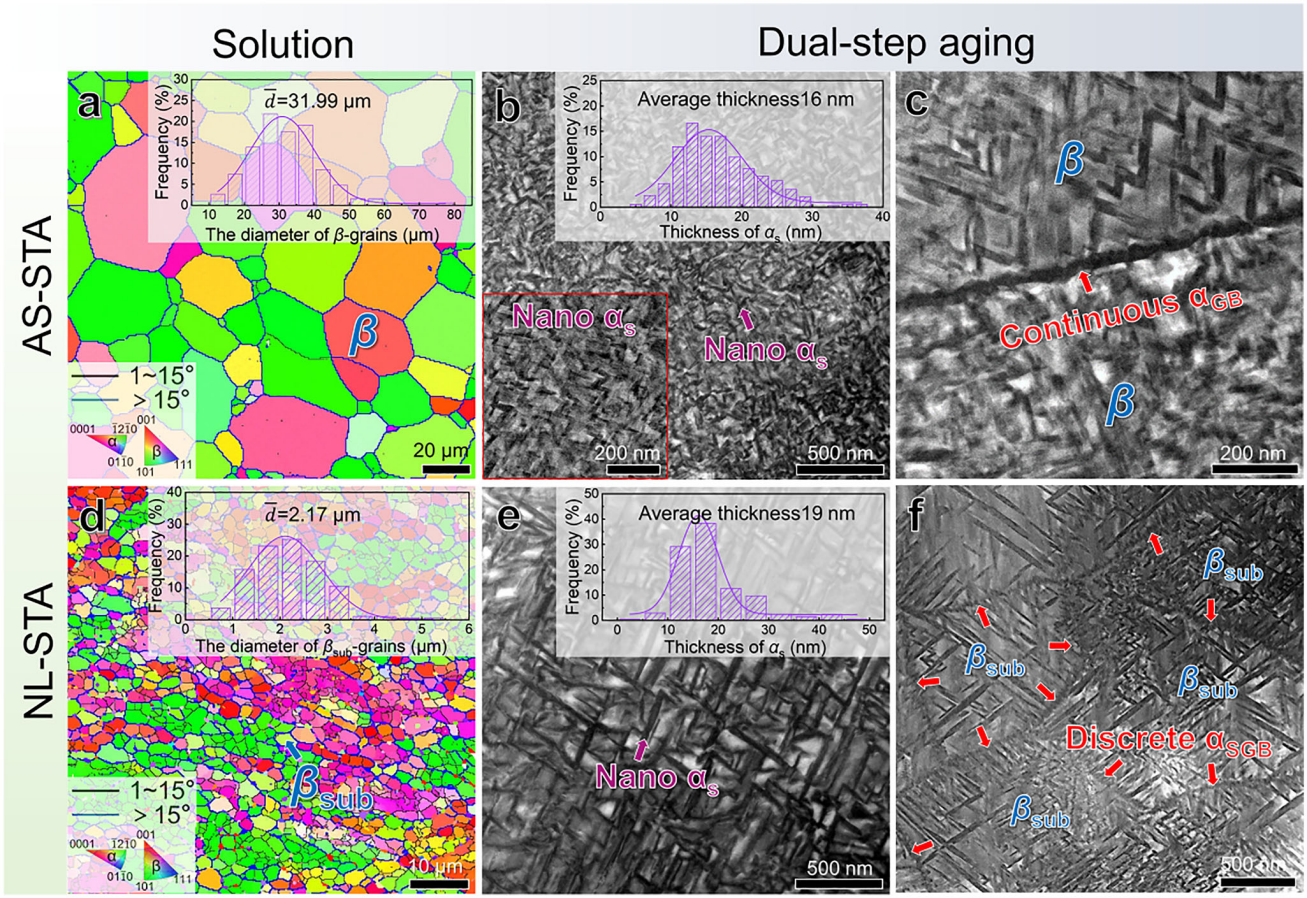
Morphological evolution of the AS‐STA and NL‐STA samples subjected to sequential solid solution and dual‐step aging treatments. a–c) correspond to the AS‐STA samples, while d–f) are associated with the NL‐STA samples. (a) An IPF map overlaid with an orientation map. (b and c) HAADF images of *β*‐grain interiors and *β*‐GBs after dual‐step aging treatment, respectively. The upper‐right insets in (a) and (b) present statistical histograms of the diameter of *β*‐grains and the thickness of *α_s_*‐nanoprecipitates, respectively. The lower‐left inset in (b) is a high‐magnification image of *α_s_*‐nanoprecipitates. (d) An IPF map overlaid with an orientation map. (e,f) HAADF images of *β*_sub_‐grain interiors and *β*‐SGBs after dual‐step aging treatment, respectively. The upper‐right insets in (d,e) are statistical histograms of the diameter of *β*_sub_‐grains and the thickness of *α_s_*‐nanoprecipitates, respectively.

For comparison, the microstructures of AS‐STA and NL‐STA samples were also investigated (**Figure** **3**). Unlike the NS‐STA sample, the solution‐treated microstructure in AS‐STA samples predominantly consists of fully recrystallized *β*‐grains decorated by HAGBs (Figure 3a). The average grain size was statistically determined to be ≈32 µm. After the dual‐aging treatment, the *β*‐grain interiors are densely populated with *α_s_*‐nanoprecipitates averaging 16 nm in thickness, while the boundaries have a continuous thick *α*_GB_‐layer, see Figure 3b,c. For the NL‐STA samples, despite the *β_sub_*‐grains being the dominant microstructural feature, their dimensions are apparently enlarged, with an average diameter of ≈2.17 µm, due to the prolonged solution duration, see Figure 3d. Upon dual‐aging treatment, the average *α_s_*‐thickness in *β_sub_*‐grain interiors is of 19 nm, whereas discontinuous *α*_SGB_ nanolaths emerge at *β*‐SGBs (Figure 3e,f).

### 2.3 Outstanding Mechanical Properties

**Figure 4:**
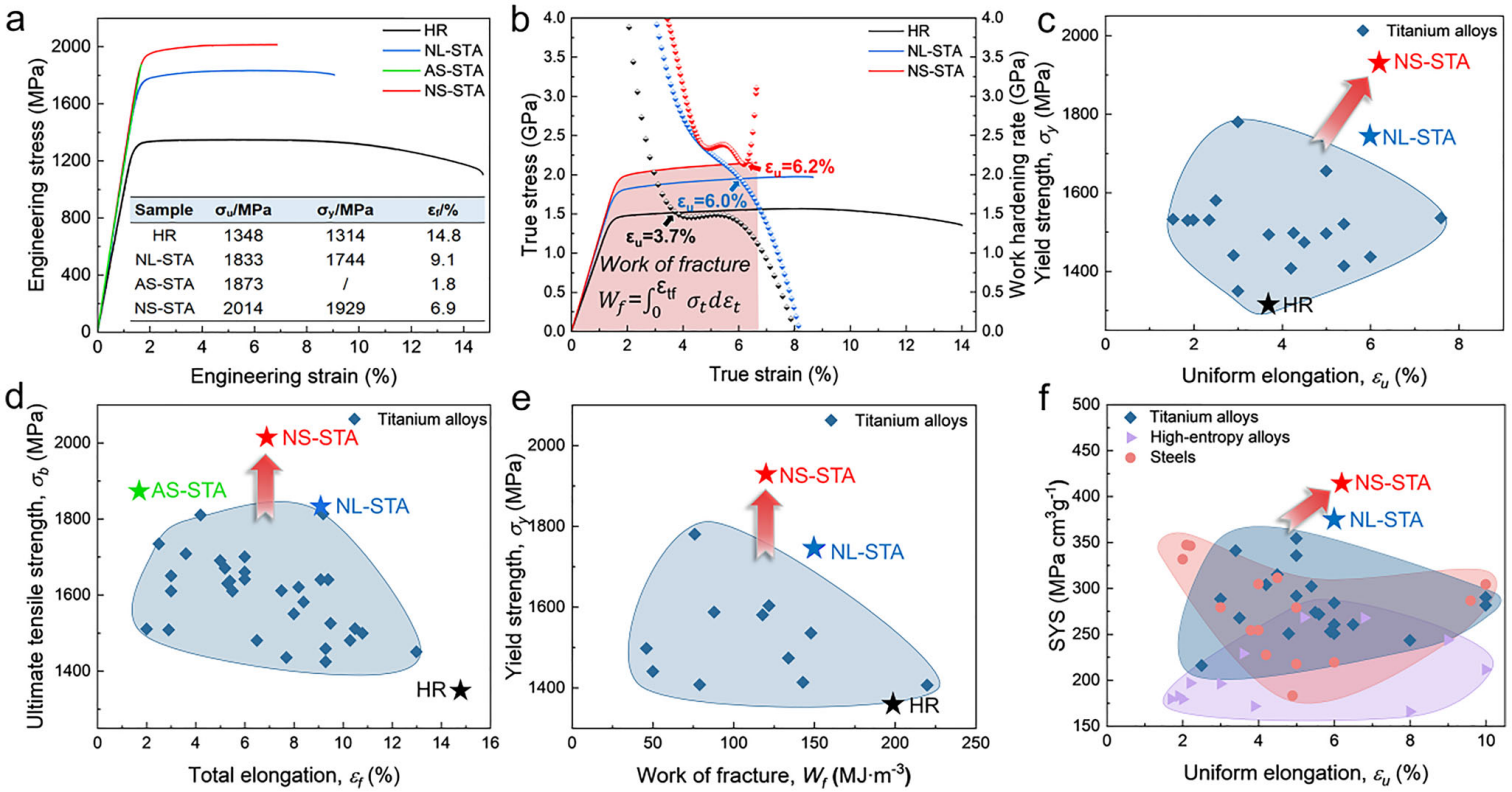
Ambient‐temperature tensile properties of the NS‐STA, NL‐STA, AS‐STA, and HR samples. a) Representative engineering stress–strain curves. The inset provides tensile data for the four types of samples. b) Corresponding true stress–strain curves and work hardening rate curves derived from (a). c–e) Comparisons of *σ_y_* vs *ε_u_*, *σ_b_* vs *ε_f_* and *σ_y_* versus *W_f_* for the current Ti alloys with those of other high‐strength Ti alloys reported in literature,^[^ ^15^, ^16^, ^17^, ^34^, ^38^, ^41^, ^42^, ^46^, ^47^ ^]^ respectively. f) Comparison of SYS vs *ε_u_* for the current alloy relative to other reported high‐strength structural materials, including Ti alloys,^[^ ^15^, ^16^, ^17^, ^34^, ^38^, ^41^, ^42^, ^46^, ^47^ ^]^ steels,^[^ ^2^, ^51^, ^52^, ^53^, ^54^ ^]^ and high‐entropy alloys.^[^ ^55^, ^56^, ^57^, ^58^, ^59^, ^60^ ^]^

**Figure** **4**a shows representative tensile engineering stress–strain curves for the NS‐STA, AS‐STA, NL‐STA, and HR Ti alloys at ambient temperature. Additional tensile curves to demonstrate testing repeatability are provided in Figure S7 (Supporting Information). It is evident that all samples exhibit smooth plastic flow without serrated fluctuation associated with Lüders or Portevin–LeChâtelier bands in ultrastrong steels.^[^ ^1^ ^]^ For the as‐received HR samples, the YS *σ_y_*, ultimate tensile strength (UTS, *σ_b_*) and ductility *ε_f_* were separately measured to be of ≈1314 MPa, ≈1348 MPa, and ≈14.8%. When *α_s_*‐nanoprecipitates are introduced into *β*‐grains, the AS‐STA samples (without SGBs) show intergranular brittle fracture at a high stress level of ≈1873 MPa during the elastic deformation stage (Figure S8, Supporting Information). When the microstructure unites *β_sub_*‐grains and *α_s_*‐nanoprecipitates, the NL‐STA samples exhibit high strength of *σ_y_* ≈1744 and *σ_b_* ≈1833 MPa, and ductility of *ε_f_* ≈9.1%. In contrast, coupling ultrafine *β_sub_*‐grains with *α_s_*‐nanoprecipitates in the NS‐STA samples, *σ_y_* and *σ_b_* are further increased to ≈1929 and ≈2014 MPa, respectively, while retaining notable ductility *ε_f_* ≈6.9% with intragranular ductile fracture (Figure S8, Supporting Information). These results indicate that an unexpected super‐strength breaking through 2 GPa can be achieved in ductile Ti alloys by devising this unique microstructure. Figure 4b shows true stress–strain curves and WHR curves of the NS‐STA, NL‐STA, and HR alloys. The NS‐STA samples exhibit multi‐stage work hardening behavior and a higher WHR compared to both NL‐STA and HR samples. The characteristics of each work hardening stage and the underlying mechanisms are analyzed in Section S2 (Supporting Information). Moreover, the NS‐STA samples show UE *ε_u_* of ≈6.2%, accounting for ≈90% of *ε_f_*. This value is higher than the *ε_u_* of ≈6.0% and ≈3.7% for the AS‐STA and HR samples, respectively. These results imply that the synergistic effect of ultrafine *β_sub_*‐grains (in particular the SGBs) and *α_s_*‐nanoprecipitates effectively delays strain localization and postpones necking.

Figure 4c,d presents the plots of *σ_y_* vs *ε_u_* and *σ_b_* vs *ε_f_* for our Ti alloys, in comparison with other high‐strength Ti alloys.^[^ ^10^, ^11^, ^15^, ^16^, ^17^, ^34^, ^36^, ^38^, ^39^, ^40^, ^41^, ^42^, ^43^, ^44^, ^45^, ^46^, ^47^, ^48^, ^49^ ^]^ Obviously, the NS‐STA samples exhibit ultrahigh strength exceeding 2 GPa while having appreciable ductility. The outstanding combination of YS and UE sets our Ti alloy apart from all previously reported high‐strength Ti alloys. We further estimated the fracture work ($W_{f}=\int_{0}^{\varepsilon_{tf}}\sigma_{t}d\varepsilon_{t}\;$) for these alloys, which generally serves as an indicator of fracture toughness and reflects the total energy required to initiate ductile failure in the fracture zone.^[^ ^50^ ^]^ The calculated *W_f_* was plotted against *σ_y_* in Figure 4e, showing the NS‐STA sample has *W_f_* ≈120 MJ m^−3^, much superior to these reported high‐strength Ti alloys. Figure 4f shows the comparison of SYS vs *ε_u_* between our Ti alloy and reported high‐strength alloys, including Ti alloys,^[^ ^15^, ^16^, ^17^, ^34^, ^38^, ^41^, ^42^, ^46^, ^47^ ^]^ steels,^[^ ^2^, ^51^, ^52^, ^53^, ^54^ ^]^ and high‐entropy alloys.^[^ ^55^, ^56^, ^57^, ^58^, ^59^, ^60^ ^]^ Still, our alloy exhibits the highest SYS combined with appreciable ductility among all the compared materials.

### 2.4 Deformation Microstructural Features

**Figure 5:**
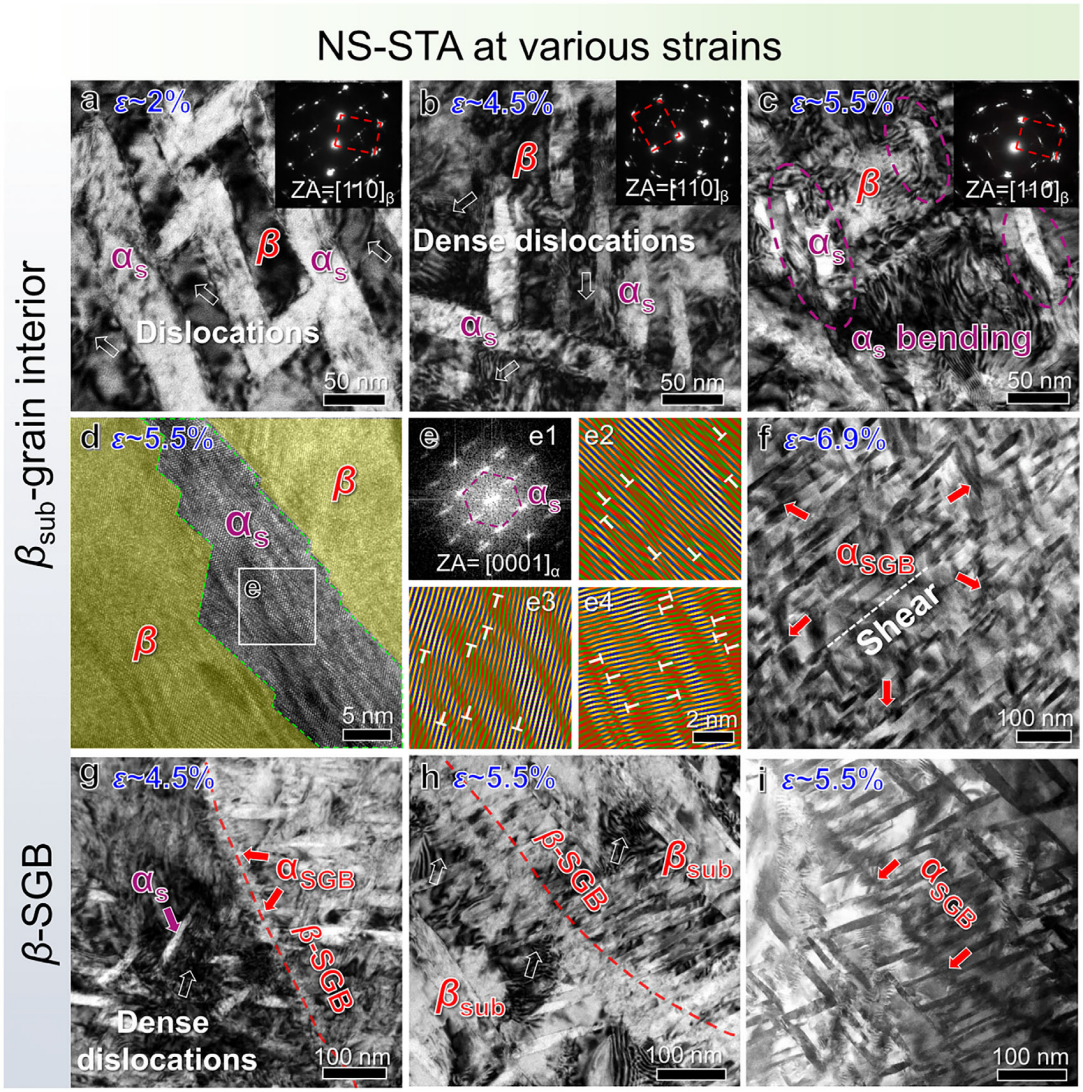
Microstructural morphologies of the NS‐STA samples after various tensile strains. a–f) are observed within the aged *β_sub_*‐grain interiors, while g–i) are located on the aged *β*‐SGBs. (a–c) TEM BF‐images under the same [110]*_β_* zone axis at strains of ≈2%, ≈4.5%, and ≈5.5%, respectively. The insets are corresponding SAED patterns. (d) HRTEM image of an *α_s_*‐nanoprecipitates under the [0001]*_α_* zone axis at a strain of ≈5.5%. (e) FFT and 1D IFFT images of the *α_s_*‐nanoprecipitates corresponding to the frame in (d). Three 1D lattice fringes were reconstructed using three sets of ${\left\langle 11\bar{2}0\right\rangle }_{\alpha }$ reciprocal spots in the FFT image. Panel “e1” shows corresponding FFT pattern, while panels “e2”, “e3”, and “e4” display the resulting 1D IFFT images obtained from different ${\left\langle 11\bar{2}0\right\rangle }_{\alpha }$ spots. (f) Low‐magnification HAADF image after tensile fracture (the strain ≈6.9%). (g) TEM BF‐image around the aged *β*‐SGBs at a large strain of ≈4.5%. (h,i) TEM BF‐image and corresponding HAADF image around the aged *β*‐SGBs at a larger strain of ≈5.5%, respectively.

**Figure** **5**a–c presents TEM BF‐images of the samples tensioned to strains of ≈2%, ≈4.5%, and ≈5.5% under the same [110]*_β_* zone axis, respectively. It is evident that gliding dislocation serves as the predominant carrier to accommodate plastic deformation, with a progressive increase in the dislocation density with straining. Individual dislocations were observed to nucleate at the *α_s_*/*β* PB, subsequently propagating into the *β*‐nanoblocks separated by these *α_s_*‐nanoprecipitates (Figure 5a). As the strain increases to 4.5%, dense dislocations continue to be activated and stored within *β*‐nanoblocks (Figure 5b). Some dislocations become entangled, while others form pile‐ups and impinge on neighboring *α_s_/β* PBs. This dislocation activity arouses new dislocation gliding in the precipitates, enabling harmonious deformation through slip transmission. Given that dense *α_s_*‐nanoprecipitates subdivide the *β*‐matrix into numerous *β*‐nanoblocks, this process occurs extensively for homogeneously macroscopical deformation. With the strain increases to 5.5%, *α_s_*‐nanoprecipitates undergo bending and even fragmentation as a result of intensive dislocation activity (Figure 5c). Consequently, the SAED pattern is featured with arc‐shaped spots, implying local variations in crystallographic orientations. Abundant dislocations were observed in the deformed *α_s_*‐nanoprecipitate (Figure 5d,e). Such plastic deformation within *α_s_*‐nanoprecipitates becomes increasingly widespread at the fracture strain ≈6.9% in Figure 5f, associated with frequent shearing events of *α_s_*‐nanoprecipitates. Consequently, the original chevron‐like *α_s_*‐pairs consisting of two *α*‐variants exhibit selective retention: only the variant aligned parallel to the shear direction is distinct, whereas the other inclined variant becomes highly ambiguous. This phenomenon suggests the occurrence of deformation‐induced elemental partitioning.^[^ ^61^ ^]^

**Figure 6:**
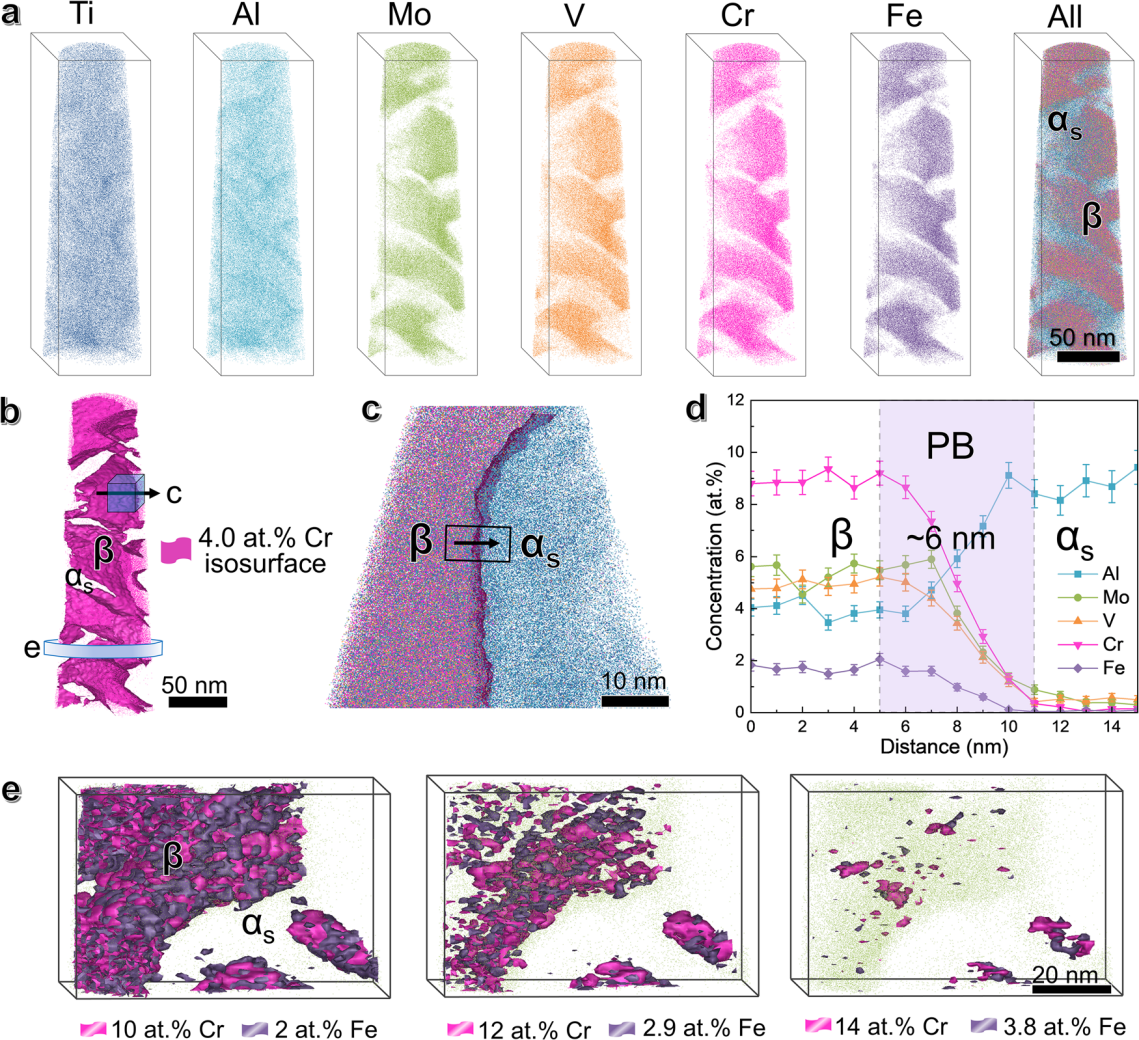
APT reconstruction and composition analysis of the NS‐STA samples after tensile fracture. a) The reconstructed APT atomic maps. b) 4.0 at.% Cr isoconcentration surface. c) An enlargement around PB between the *α_s_*‐nanoprecipitate and the *β*‐matrix along the arrow in (b). d) Corresponding composition profile of Mo, V, Fe, Cr, and Al atoms measured across the arrow in (c), showing the interfacial thickness of 6 nm. e) Various iso‐concentration surfaces of *β*‐matrix interior taken from the circle slice in (b), showing the enhanced chemical heterogeneities of Cr and Fe enriched regions along with the presence of elongated decoration zones caused by segregation to dislocations.

Indeed, APT reconstruction in **Figure** **6**a confirms that *β*‐stabilizers such as Mo, V, Cr, and Fe begin to diffuse with reduced concentrations, rather than initially concentrated segregation in the *β*‐phase (Figure 2). Moreover, the PBs between *α_s_*‐nanoprecipitates and *β*‐nanoblocks in Figure 6b become noticeably curved in shape. The *α_s_*/*β* PB region shows an intermixed layer of alloying atoms with an increased thickness of ≈6 ± 1 nm, see Figure 6c,d. The 2D in‐plane atomic distribution maps in Figure S5 (Supporting Information) provide a more visual illustration of element diffusion at the *α_s_*/*β* interfaces. This elemental partitioning is probably correlated with dense dislocations that transmit across the *α_s_*/*β* interfaces, as dislocations often serve as fast diffusion pathways and can drag atom motion under high applied stress (2 GPa).^[^ ^61^ ^]^ Figure 6e shows more pronounced local composition inhomogeneities after tension, in particular Cr‐ and Fe‐enriched nanoscale regions within the *β*‐matrix. Linear features are revealed by isoconcentration envelopes surrounding zones in the data containing a high concentration of Cr (14 at.%) and Fe (3.8 at.%), indicating their segregation along dislocation lines. This suggests that strong interactions between Cr or Fe atoms and dislocation cores enhance chemical heterogeneities in the *β*‐nanoblocks during plastic straining.^[^ ^61^ ^]^ Such segregation behavior is further verified by the reconstructed atom maps in Figure S6 (Supporting Information). The deformation‐induced elemental partitioning is also theoretically supported. For more details, please refer to Section S3 (Supporting Information).

Regarding the discontinuous *α*_SGB_ at *β*‐SGBs, they also serve as dislocation sources for plasticity at the *α_s_*/*β* interfaces. This aged *β*‐SGB structure confines motion of dislocations generated from *β_sub_*‐grain interiors. For instance, high‐density dislocations pile‐up associated with notable strain contrast near the aged *β*‐SGBs at a strain of ≈4.5% (Figure 5g). As a result, these *β*‐SGBs are less distinguishable due to intensive dislocation activities. Upon increasing the strain to ≈5.5%, the *α*_SGB_ begins to suffer from shearing, enabling dislocations to glide into adjacent *β_sub_*‐grain for further plastic deformation (Figure 5h). Figure 5i displays a corresponding HAADF image that filters out chaos strain contrast. It is apparent that the aged *β*‐SGB exhibit a curved morphology caused by homogeneous strain partitioning due to frequent slip transfer. Taken together, the aged ultrafine *β_sub_*‐grains provide more extensive plastic deformation capability, leading to the improved tensile uniform elongation, compare with SGB‐free even NL‐STA samples.

## 3 Discussion

### 3.1 Mechanisms for Ultrahigh Yield Strength

A superior synergy of record‐high YS and appreciable UE has been achieved in the present NS‐STA Ti alloys (Figure 4). The ultrahigh strength (*σ_y_* ≈1929 MPa and *σ_b_* ≈2014 MPa) sets the current Ti‐4Al‐5Mo‐3V‐5Cr‐1Fe alloy apart from previous high‐strength Ti alloys. The dominant strengthening mechanism responsible for this exceptional strength can be attributed to precipitation strengthening contributed by intragranular *α_s_*‐nanoprecipitates, as well as precipitation strengthening arising from intergranular *α_SGB_*‐nanolaths and SGB strengthening originating from *β*‐SGBs within ultrafine *β_sub_*‐grains. Upon loading, dislocation activity is strongly impeded inside the complex 3D multi‐scale hierarchical structure.^[^ ^1^, ^62^, ^63^ ^]^ Consequently, an ultra‐high stress is required for yielding the NS‐STA alloys.

To elucidate the contributions of precipitation strengthening from intragranular *α_s_*‐nanoprecipitates(*σ_α_*_s_) and intergranular *α_SGB_*‐nanolaths (*σ_α_*_SGB_), as well as SGB strengthening (*σ_β‐sub_*) from ultrafine *β_sub_*‐grains to the overall YS of the NS‐STA alloys, they are evaluated alongside other strengthening components, including solid solution strengthening (*σ*_0_) and Taylor strengthening (*σ*_ρ_). The calculated YS (*σ*_y_), obtained by summing these five contributors, is ≈1951 MPa, which agrees well with the experimentally measured value ≈1929 MPa (Figure 4a). Notably, *σ_α_*_s_, *σ_α_*_SGB_, and *σ_β‐sub_* are estimated to be 965, 33, and 257 MPa, respectively. Their combined contribution amounts to 1255 MPa, accounting for ≈64% of the overall yield strength. For further details, please refer to the theoretical calculation of YS provided in Section S4 (Supporting Information).

### 3.2 Mechanisms for Appreciable Uniform Elongation

In Ti alloys, the enhanced UE is typically associated with mechanical twinning or deformation‐induced phase transformation via the well‐known TWIP and/or TRIP effects.^[^ ^50^, ^64^ ^]^ However, only ordinary dislocation gliding was detected in the current Ti alloy during plastic deformation. This indicates that plasticity is primarily accommodated by ordinary dislocation gliding, with dislocation activity uniquely regulated to achieve both sustained work hardening and high uniform ductility.

Upon straining, apart from *α_s_*/*β* PBs acting as dislocation sources, SGBs also generate abundant dislocations.^[^ ^20^, ^21^, ^23^, ^24^ ^]^ Once the local stress reaches the critical resolved shear stress for dislocation gliding within *α_s_*‐nanoprecipitates, dislocation slip is activated and can transmit across the PB into the *β*‐matrix, rendering plasticity transfer via slip “relaying” from one phase to another.^[^ ^65^ ^]^ More specifically, the *α_s_*‐nanoprecipitates typically form chevron‐like pairs consisting of two *α*‐variants, which exhibit a twinning relationship and are bounded by coherent two boundaries (CTBs) (Figure S4, Supporting Information). Such twinned *α_s_*‐nanoprecipitates promote favorable plastic compatibility and slip continuity.^[^ ^10^, ^12^, ^62^ ^]^ Dislocations are capable of cutting across the twin boundary and transmitting into adjacent *α_s_*‐nanoprecipitates.^[^ ^66^ ^]^ Stress concentrations at dislocation‐CTB intersections can be effectively relieved, thereby mitigating localized stress buildup caused by dislocation pile‐up within intragranular *α_s_*‐nanoprecipitates.^[^ ^12^ ^]^

With increasing strain, a high density of aged *β*‐SGBs can hinder dislocation motion and trap dislocations into isolated regions (Figure 5), leading to more homogeneous partitioning of plastic strains throughout the microstructure. These dislocations, temporarily trapped at SGBs, can depin under sufficiently high stresses and transmit into adjacent *β_sub_*‐grains. This results in extensive dislocation‐SGB interactions, contributing to high WHRs and thus notable UE. The deformation accommodation mechanism in the NS‐STA samples is schematically illustrated in Figure S12 (Supporting Information). Both dislocation self‐interactions and interactions with dense *α_s_*/*β* interfaces or *β‐*SGBs contribute to the elevated WHRs. Furthermore, deformation‐induced composition heterogeneities in the form of Fe and Cr segregation create a unique “nano‐cocktail” environment in the NS‐STA samples, which delivers a nanoscale segment detrapping mechanism for dislocation motion.^[^ ^61^ ^]^ This renders dislocation glide in a sluggish manner, increasing the likelihood of dislocation interactions and entanglement. The progressive accumulation of dislocations dynamically introduces obstacles against ensuing dislocation movement, thereby maintain a sustained WHR.^[^ ^61^ ^]^ Consequently, plastic instability is postponed, enabling large UE in the NS‐STA Ti alloys.

## 4 Conclusion

We develop a new Ti‐4Al‐5Mo‐3V‐5Cr‐1Fe alloy with exceptional combinations of ultra‐high strength (*σ_y_* ≈1929 and *σ_b_* ≈2014 MPa) and notable uniform elongation (*ε_u_* ≈6.2%). This outstanding strength‐ductility synergy is achieved by engineering a unique microstructure that comprises intragranular dense *α_s_*‐nanoprecipitates dispersed inside ultrafine *β_sub_*‐grains decorated with intergranular discrete *α_SGB_*‐nanolaths. The multifunctional SGBs not only act as dislocation obstacles/sources for strengthening/ductilizing alloys, but also serve as diffusion promoters for nanoprecipitation and chemical heterogeneities for ultrahigh strength, enhanced WHRs and thus large UE. This strengthening‐ductilizing strategy through SGB‐mediated nanoprecipitation and chemical heterogenization is also applicable to other metallic materials such as Al alloys, Mg alloys, steels and multi‐principal element alloys, in particular those prepared via additive manufacturing to obtain dislocation substructures for advanced performances.

## 5 Experimental Section

### Material Preparation

In this study, a near *β*‐transus high‐temperature short‐term solution plus dual‐step aging route was proposed to engineer ultrafine *β_sub_*‐grains and nanoscale *α_s_*‐precipitates in Ti‐4Al‐5Mo‐3V‐5Cr‐1Fe (wt.%) alloy wires. To achieve the ultrafine *β_sub_*‐grains, an industrially feasible thermomechanical processing route was applied on the alloy with the aid of Xinjiang Xiangrun New Materials Technology Co., Ltd., China. This process involved high‐temperature forging and multi‐pass diameter reduction to refine the primitively coarse cast grains as follows: First, alloy ingots with a diameter of Ф 650 mm was prepared by double vacuum arc remelting (VAR) method. Subsequently, the ingots were heated to ≈1150 °C and forged three times to form rods with a reduced diameter of Ф 180 mm. The rods then underwent hot rolling, which included roughing, pre‐finishing, and finishing stages. During roughing rolling, the rods were heated to 950 °C and held for 120 min. They were then rolled eleven times, reducing their diameter to Ф 65 mm (equivalent to ≈87% of total deformation). For pre‐finishing rolling, the rods were reheated to 890 °C and held for 60 min. Then they were rolled to produce wires with a diameter of Ф 12 mm (equivalent to ≈99% of total deformation). Finally, these Ф 12 mm wires undertaken the finishing rolling at 790 °C for eight passes, reducing their diameter to Ф 7 mm (equivalent to ≈100% of total deformation), see their actual photo in Figure S1 (Supporting Information). These thermomechanical processes not only effectively break up the cast grains, but also introduced a large amount of deformation energy, which was primarily stored as dislocations in hot‐rolled (HR) wires. The stored deformation energy will provide a driving force for static recover and recrystallization during subsequent heat treatment. Then, the HR wires were subjected to solution treatment. It should be emphasized that the wires were classified into three types: NS, NL, and AS, based on solution temperature and treatment duration. Subsequently, these wires underwent the same dual‐step aging to precipitate *α_s_*‐precipitates, which were designated as NS‐STA, NL‐STA, AS‐STA wires, respectively.

### Measurements of Mechanical Properties

The four types of alloy wires, treated via HR, NS‐STA, NL‐STA, and AS‐STA processes, were machined into dumbbell‐shaped tensile samples with a gauge portion of 3 mm in diameter and 15 mm in length along the rolling direction (RD). Uniaxial tensile tests were conducted on an INSTRON 1195 universal testing machine at ambient temperature with a strain rate of 5 × 10^−4^ s^−1^. To ensure data reliability, all mechanical tests was repeated multiple times for each condition. Tensile properties, including yield strength (YS, *σ_y_*), ultimate tensile strength (UTS, *σ_b_*), total elongation to failure (*ε_f_*) and uniform elongation (UE, *ε_u_*), were recorded.

### Microstructural Characterizations

Prior to microstructural characterization, all samples were mechanically ground using a series of SiC abrasive papers to eliminate the influence of electrical discharge machining during the cutting process. Subsequently, they were polished for 15 s in a solution of 10% HClO_4_ and 90% CH_3_COOH (vol%). The ground samples were etched in a Kroll's reagent of 1% HF, 3% HNO_3_, and 96% H_2_O (vol%). Microstructures were characterized by scanning electron microscopy (SEM, Hitachi SU6600). *β_sub_*‐grain sizes were determined using Oxford electron backscatter diffraction (EBSD) detector mounted on a field‐emission scanning electron microscope (SEM, Zeiss GEMINI 500). The corresponding EBSD analysis relies on Channel 5 software. The thickness of *α_s_*‐precipitates imaged by transmission electron microscopy (TEM, JEOL JEM‐F200) was measured by the linear intercept method using image‐pro software. Substructural features were characterized using TEM techniques, including HRTEM and HAADF mapping, operated at 200 KV. Chemical compositions were analyzed using atom probe tomography (APT) on needle shaped specimens which were obtained by lift‐outs and annular milled in a FEI Scios focused ion beam/scanning electron microscope (FIB/SEM). Then the APT samples were characterized by a CAMECA local electrode atom probe (LEAP 4000XSi) under a high vacuum (Pa) at 20 K. The relevant data were collected and then analyzed by the Integrated Visualization and Analysis Software (IVAS) version 3.6.8 for 3D reconstructions and compositional analyses.

## Conflict of Interest

The authors declare no conflict of interest.

## Supporting information



Supporting Information

## Data Availability

The data that support the findings of this study are available from the corresponding author upon reasonable request.
