# Supplementary material for: Designing Ductile 2‐GPa Yielding Titanium Alloys via Multifunctional Subgrain Boundaries and Nanoprecipitates
Source: Adv Sci (Weinh). 2025 Nov 30;13(8):e19918. doi: 10.1002/advs.202519918 (PMC12884723; doi:10.1002/advs.202519918)
Supplement: Supplementary file 1 — Supporting Information [file ADVS-13-e19918-s001.docx]

Supporting Information

**Designing ductile 2-GPa yielding titanium alloys via multifunctional subgrain boundaries and nanoprecipitates**

Dingxuan Zhao, Kai Zu, Xu Yue, Hang Zhang, Zexuan Li, Keer Li, Zehua Zheng, Jialuo Yang, Wei Chen*, Jinyu Zhang*, Jun Sun

This supporting information file includes:

Supplementary Text

Figures. S1 to S12

Table S1

Additional references

**Supplementary Text.**

**Section S1.** **Discussion on local chemical heterogeneities in the NS-STA samples prior to tension**

High-entropy alloys (HEAs), as representative multi-component complex materials, are inherently prone to compositional inhomogeneity.^[1, 2]^ The magnitude of compositional fluctuations in these alloys significantly exceeds that observed in conventional dilute solutions. This phenomenon occurs because, when the overall composition is close to the center of a multi-component system, the standard deviation of elemental concentrations increases substantially.^[2]^ Consequently, local regions exhibit concentration variations on the order of several atomic percent. From a thermodynamic standpoint, such local chemical heterogeneities in HEAs originate from high positive mixing enthalpies, which counteracts configurational entropy and promotes elemental de-mixing.^[3]^

In this study, our Ti-4Al-5Mo-3V-5Cr-1Fe alloys also belong to the class of multi-component complex materials. The APT analysis in Figure 2 reveals pronounced local chemical heterogeneities prior to deformation. These segregation phenomena can be rationalized by examining the thermodynamic interaction parameters among the constituent binary element pairs. The key intrinsic factors governing element segregation are the mixing enthalpy and relative binding energies. Elements with positive mixing enthalpies and high relative binding energies tend to segregate. Reported data indicate that the Ti-Fe and Ti-Cr binary systems exhibit high positive mixing enthalpies (+33.9 kJ/mol and +7.5 kJ/mol, respectively), while the Fe-Fe (471 MeV) and Cr-Cr (411 MeV) binding energies exceed that of Ti-Ti (375 MeV).^[4, 5]^ Clearly, Fe and Cr possess favorable thermodynamic driving forces for solute clustering within the *β*-matrix. Moreover, both Fe and Cr are *β*-stabilizing elements and belong to the *β*-eutectoid forming group.^[6]^ They are capable of forming intermetallic compounds via eutectoid reactions, for which elemental segregation provides the necessary compositional precondition. This may constitute an additional mechanism contributing to the observed local chemical heterogeneities of Fe and Cr in the alloy, as shown in Figure 2e.

**Section S2. Analysis of multi-stage work hardening behaviors**

The work hardening rate curves, together with the corresponding true stress-strain curves of the NS-STA and NL-STA samples, are extracted from Figure 4b and presented in Figure S9 (Supporting Information). With increasing strain, four distinct work hardening stages, labeled as Ⅰ-Ⅳ, are clearly identifiable in the NS-STA samples. Stages Ⅰ and Ⅲ are characterized by a continuously decreasing work hardening rate. Stage Ⅱ exhibits a gradually increasing work hardening rate, whereas Stage Ⅳ demonstrates a sharply elevated work hardening behavior. Combining TEM observations of the deformation microstructures at representative tensile strains in Figure 5, the underlying mechanism responsible for this multi-stage work hardening behavior can be elucidated.

When the NS-STA samples initially undertake plastic deformation upon loading, dislocations preferentially nucleate from *α_s_*/*β* PBs within *β_sub_*-grain interiors and from discontinuous *α*_SGB_ at *β*-SGBs. The subsequent relaxation of stress by these early-formed dislocations during the incipient plastic stage leads to the observed continuous decrease in work hardening rate, corresponding to Stage Ⅰ. As strain increases, more dislocations are activated at these dislocation sources and propagate until impeded by the *α_s_*/*β* PBs, resulting in dislocation piling-up. This phenomenon becomes increasingly pronounced due to the high-density precipitation of *α_s_* and *α*_SGB_ nanolaths in the aged microstructure. Within the resulting constrained spaces, dislocations undergo strong interactions and multiplication, contributing to the gradually rising work hardening rate observed in Stage Ⅱ. The accumulation of dislocations and their interactions induce stress concentration at the *α_s_*/*β* PBs. Once the concentrated stress reaches the critical resolved shear stress (CRSS) required for dislocation gliding in the neighboring *α_s_*-phase, plastic deformation initiates within this phase through the activation of new dislocations or via slip transmission across the *β*/*α_s_* PBs. With further straining, this deformation transfer even evolves into shearing and fragmentation of the *α_s_*-laths, leading to a partial release of accumulated internal stress and thus accounting for the second decrease in work hardening rate (Stage Ⅲ). Nevertheless, such strain localization does not immediately lead to uncontrolled deformation under continued loading. The propagation of deformation or shearing bands is effectively hindered upon encountering the aged *β*-SGBs. This obstruction promotes renewed dislocation piling-up and multiplication within the *β_sub_*-grain interiors, thereby corresponding to the second increase in work hardening rate (Stage Ⅳ). Eventually, when sufficient stress accumulates to overcome the resistance of the aged *β*-SGBs, uncontrollable long shearing band quickly form and traverse the microstructure, resulting in final fracture of the NS-STA samples.

By contrast, the work hardening rate curve of the NL-STA samples can be divided into three distinct stages, labelled as Ⅰ-Ⅲ in Figure S9 (Supporting Information). Stages Ⅰ and stage Ⅲ are featured by a continuously decreasing work hardening rate, while Stage Ⅱ exhibits a slowly decreasing work hardening behavior. Similar to the NS-STA samples, the NL-STA samples initiate plastic deformation through dislocation activation at *α_s_*/*β* PBs within *β_sub_*-grain interiors and discontinuous *α*_SGB_ at *β*-SGBs. Therefore, work hardening rate decreases gradually with increasing strain, corresponding to Stage Ⅰ. However, a notable structural difference in the NL-STA samples is the coarsening of *β*-SGBs with an average diameter 2.17 μm, approximately three times larger than that of 613 nm in the NS-STA samples. This grain growth leads to a global reduction in the number of precipitated *α*_SGB_ nanolaths in the aging microstructure, thereby decreasing the density of available dislocation sources for nucleation. As a result, the work hardening rate during Stage Ⅰ in the NL-STA samples remains consistently lower than that in the NS-STA counterparts. Furthermore, as dislocations propagate and accumulate ahead of the *α_s_*/*β* PBs, the resulting pile-up effect is also pronounced less compared to that in the NS-STA samples. Consequently, this only moderates the rate of decline in work hardening rate (Stage Ⅱ), rather than inducing a reversal into an increasing trend like that in the NS-STA samples. Furthermore, the coarsened *β*-SGBs possess a weak capacity to impede the propagation of shear bands within the *β_sub_*-grain interiors. Therefore, stage Ⅲ in the NL-STA samples continues to display a monotonically decreasing work hardening rate, in contrast to the sharp increase in Stage Ⅳ of the NS-STA samples.

**Section S3. Discussion on deformation-induced elemental partitioning in the NS-STA samples**

TEM imaging and APT analysis indicates that elemental partitioning caused by atomic diffusion occurs within the deformed NS-STA samples (Figures 5 and 6). This elemental partitioning is associated with dense dislocations that transmit across the *α_s_*/*β* interfaces, as dislocations can serve as diffusion path and drag atom motion under a high stress.^[7-9]^ Furthermore, the diffusion distance was estimated to be ~3 nm based on the difference in the intermixed layer thickness of alloying atoms within the the *α_s_*/*β* PB regions before and after tensile deformation (Figures 2d and 6d). Such deformation-driven atomic diffusion under high stress can be theoretically justified and the resulting diffusion distance is quantitatively derived as follows:

Atomic diffusion at ambient temperature in normal metals mainly occurs via short-circuit diffusion, which are mediated by structural defects such as grain boundaries and dislocations.^[10]^ In defect-free bulk metals, the diffusion coefficient *D_bulk_* can be described by the Arrhenius equation:

$D_{Bulk}=D_{0}exp\left( \frac{-Q_{Bulk}}{RT} \right)$ (1)

where *D_0_* is frequency factor, *Q_Bulk_* is the activation energy for diffusion, *R* is the gas constant, and *T* is the applied temperature. At ambient temperature, the *Q_Bulk_* remains relatively high, resulting in negligible diffusion rates in bulk metals under free-stress condition. However, during plastic deformation, dislocation-assisted short-circuit diffusion becomes significant, with the activation energy (*Q_dis_*) estimated to be ~0.57-0.74 times *Q_bulk_*.^[11]^ This indicates that dislocations substantially reduce the energy barrier for atomic migration, thereby enhancing diffusivity. Moreover, since dislocations are activated by applied stress during deformation, the influence of mechanical stress must be incorporated into the diffusion model. A modified expression accounting for the effect of applied stress is given by:^[12]^

$Q_{eff}=Q_{bulk}-\tau\Omega$ (2)

where *Q_eff_* represents the effective activation energy, *τ* is the shear stress associated with the externally applied stress, and *Ω* is the strain volume. Hence, *Q_eff_* functions as a thermodynamic potential dependent on both *T* and *τ*. The stress-assisted diffusion behavior through dislocation pathways under stress can be thus expressed as:^[11]^

$D_{eff-dis}=D_{0-dis}exp\left( -\frac{Q_{eff}}{kT} \right)=D_{0-dis}exp\left( -\frac{Q_{Bulk}-\tau\Omega}{kT} \right)$ (3)

where *D_0-dis_* is frequency factor of atomic diffusion through dislocation channel, and *D_eff-dis_* denotes the diffusion coefficient for stress-assisted atomic diffusion along dislocation channel. Eq. (3) demonstrates that *D_eff-dis_* exceeds *D_bulk_*, implying enhanced diffusivity under applied stress. As a result, the diffusion distance *L* achieved via dislocation pathways is greater than that of bulk diffusion. The *L* can be estimated according to:^[13]^

$L=\sqrt{D_{eff}t}$ (4)

where *t* represents the duration of the loading process, *i.e.*, the effective diffusion time under stress.

Regrettably, due to the absence of corresponding parameters for *D_0-dis_* and *Q_Bulk_* in the current Ti-4Al-5Mo-3V-5Cr-1Fe alloys at ambient temperature, the exact value of *L* under stress conditions can be not readily determined. Here, we provide only a rough estimation based on available stress-free diffusion data. The temperature-dependent diffusion behavior of Al atoms in BCC *β-*phase is extrapolated to ambient temperature using reported data,^[13]^ yielding an estimated *D*, as illustrated in Figure S10 (Supporting Information). Consequently, the value of *D* is deduced to be ~10^-20^ m^2^∙s^-1^. The duration of the tensile test is assumed to be ~200 s. Accordingly, the *L* is thus calculated to be ~1.4 nm. Although the value is smaller than the 3 nm measured by the APT analysis, it remains reasonable because the actual *D_eff-dis_* under high applied stress is expected to be higher than the stress-free *D*.

**Section S4. Theoretical calculations of ultrahigh strength**

The NS-STA samples have demonstrated a superior combination of ultrahigh strength and considerable ductility. The ultrahigh yield strength (*σ_y_* ~1929 MPa) is attributed to the synergistic combinations of solid solution strengthening (*σ_0_*), subgrain boundary strengthening (*σ_β-sub_*), Taylor strengthening (*σ_ρ_*), intragranular *α_s_* precipitation strengthening (*σ_αs_*), and intergranular *α_SGB_* precipitation strengthening (σ*_αSGB_*). This ultrahigh strength *σ_y_* is quantitatively rationalized by summing these individual strengthening components according to the mixing rule, as expressed in the following equation:^[14]^

$$\sigma_{y}=\sigma_{0}+\sigma_{\beta-sub}+\sigma_{\rho}+\sigma_{\alpha SGB}{+\sigma}_{\alpha s} (1)$$

Here, *σ_0_* represents the lattice friction stress. For the current alloy, *σ_0_* can be regarded as the sum of the critical resolved shear stress of pure Ti (*σ_CRSS_* ~180 MPa)^[15]^ and solid-solution strengthening (*σ_SS_*), given by:

$$\sigma_{0}=\sigma_{CRSS}+\sigma_{SS} (2)$$

$${\sigma_{SS}=\left( \sum_{i} B_{i}^{\frac{3}{2}}X_{i} \right)}^{\frac{2}{3}} (3)$$

where *X_i_* and *B_i_* are the atomic fraction of solute elements and strengthening coefficient, respectively. The values of *B_i_* are obtained from the literature,^[15]^ and depend on the mismatch parameter (*λ_i_*) between each substituent element and Ti atoms. Relevant parameters are listed in Table S1. Using these data, *σ_0_* is estimated to be ~479 MPa.

With regard to *β*-subgrain boundary strengthening (*σ_β-sub_*), owing to the unique ultrafine *β_sub_*-grain structure in the NS-STA samples, an attempted was made to quantify *σ_β-sub_* based on the influence of LAGBs. The quantitative expression is as follows:^[16]^

$$\sigma_{\beta-sub}=M\alpha Gb\left( \frac{3\left( 1-f_{HAGB} \right)\theta_{mean}^{LAGB}}{bL} \right)^{\frac{1}{2}} (4)$$

Where *M* = 2.8 is the Taylor factor, *α* ~0.3 is the dislocation interaction constant depending on crystal structure, *G* = 39 GPa is the shear modulus of the *β*-phase, *b* = 2.8 $Å$ is the magnitude of the Burgers vector of dislocations in the *β*-phase, *f_HAGB_* is the fraction of HAGBs (∼6 % in Fig. 1b), $\theta_{mean}^{LAGB}$ is the average misorientation angle of LAGBs (∼2.73° in Fig. 1b), and *L* is the average *β*-subgrains size (∼613 nm in Fig. 1a). Based on these parameters, *σ_β-sub_* is calculated to be ~257 MPa.

Taylor strengthening (*σ_ρ_*) is described by the classical Taylor hardening relation:^[17]^

$$\sigma_{\rho}=\alpha MGb\sqrt{\rho} (5)$$

where *ρ* is the dislocation density, which can be determined using the following equation:^[13]^

$$\rho=\frac{2\theta_{KAM}}{Xb} (6)$$

with *X* ~0.25 μm being the step size. *θ_KAM_* represents the average KAM values, derived from the formula:^[13]^

$$\theta_{KAM}=\sum f_{i}\theta_{i} \theta<5^{^{\circ}} (7)$$

where *θ_i_* and *f_i_* denote the local misorientation and its corresponding frequency, respectively.

Based on the KAM analysis in Fig. S11, the average KAM values for the NS-STA samples is measured to be ~1.12°. By combining Eqs. (5)-(7), the dislocation density is estimated to be ~5.585×10^14^ m^-2^ and thus *σ_ρ_* is calculated to be ~217 MPa.

Give that intergranular *α*_SGB_ nanolaths precipitated at *β*-SGBs impede dislocations motion, their contribution to strength can be estimated using the following model:^[18]^

$\sigma_{\alpha SGB}=0.5f_{\alpha SGB}\left( \sigma_{0}+\sigma_{\beta-sub}+\sigma_{\rho} \right)$ (8)

where *f_αSGB_* is the volume fraction of intergranular α_SGB_ nanolaths. According to TEM characterization in Figure 1g, *f_αSGB_* is measured to be ~7%. Substituting the previously calculated values of *σ_0_*, *σ_sub_* and *σ_ρ_* (479 MPa, 257 MPa and 217 MPa, respectively), the contribution of *α_SGB_* is estimated to be ~33 MPa.

Finally, precipitation hardening (*σ_α_*_s_) arising from intragranular *α_s_*-nanoprecipitates is expressed as:^[19]^

$$\sigma_{\alpha s}=\frac{Gb}{2\sqrt{2}}\frac{n}{h} (9)$$

where *n* is the number of dislocations crossing the *α*-plates (∼4 in Fig. S11), and *h* is the average thickness of the *β*-matrix (∼16 nm in Fig. S4a). Benefited from the formation of *α_s_*-nanoprecipitates, *σ_αs_* is calculated to reach 965 MPa. Consequently, the theoretical calculation (Cal.) of *σ_y_* = 1951 MPa shows good agreement with the experimental measurement (Exp.) *σ_y_* = 1929 MPa for the the NS-STA samples, as illustrated in Fig. S11.

**Supplementary Figures**


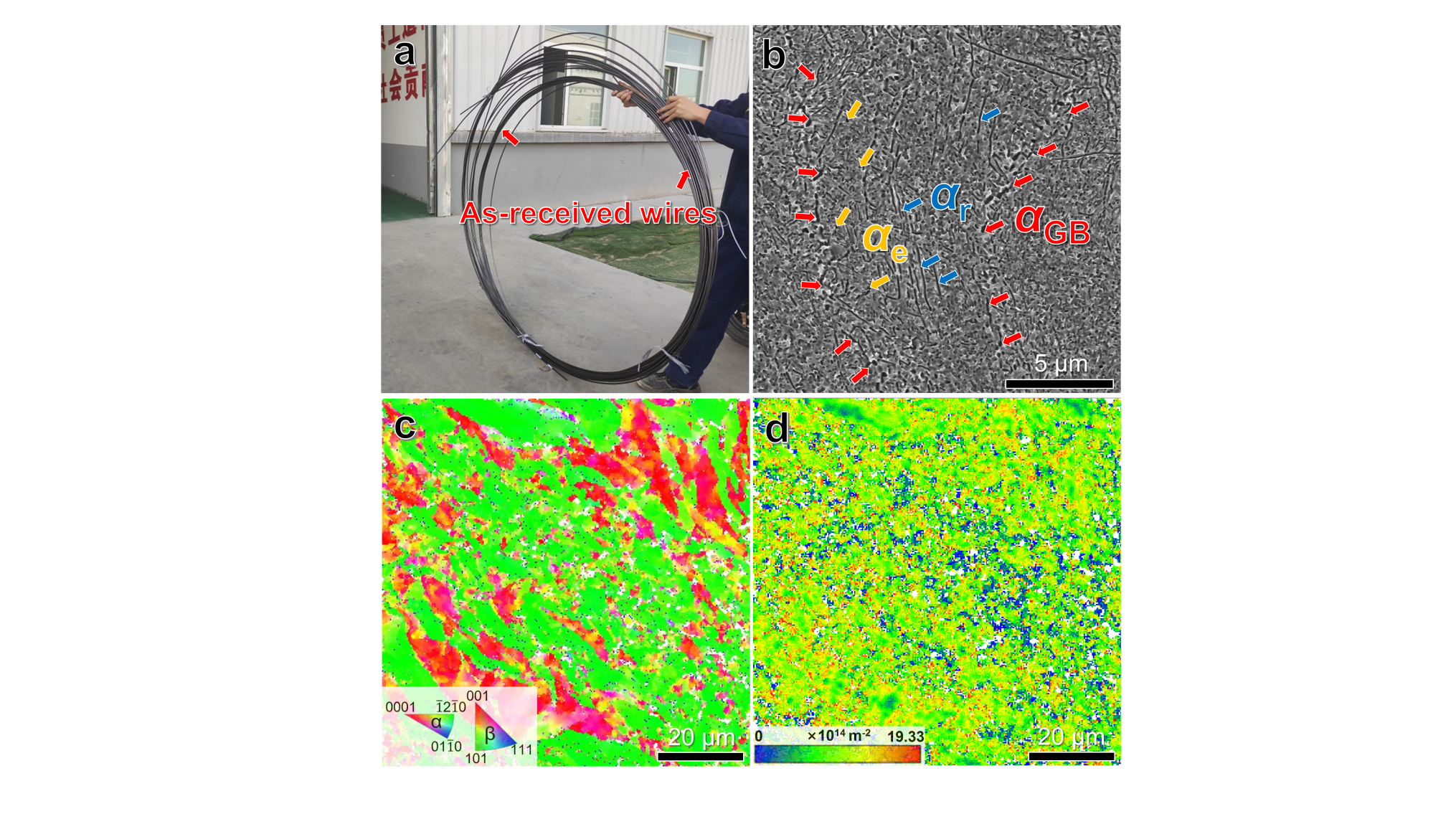


**Figure. S1.** The as-received hot-rolled Ti-4Al-5Mo-3V-5Cr-1Fe titanium alloy wires and their microstructural morphologies. (a) The product of as-received wires. (b) The SEM image showing dramatic microstructural fragmentation due to hot rolling. Equiaxed *α* (*α*_e_), rod-shape *α* (*α*_r_) and dispersive grain-boundary *α* (*α*_GB_) with large strain distortion distribute within the microstructure. (c) The EBSD inverse pole figure (IPF) showing frequent color variations as a result of large rolling deformation. (d) The geometrically necessary dislocation (GND) map showing abundant dislocations preserved in the hot-rolled microstructure.


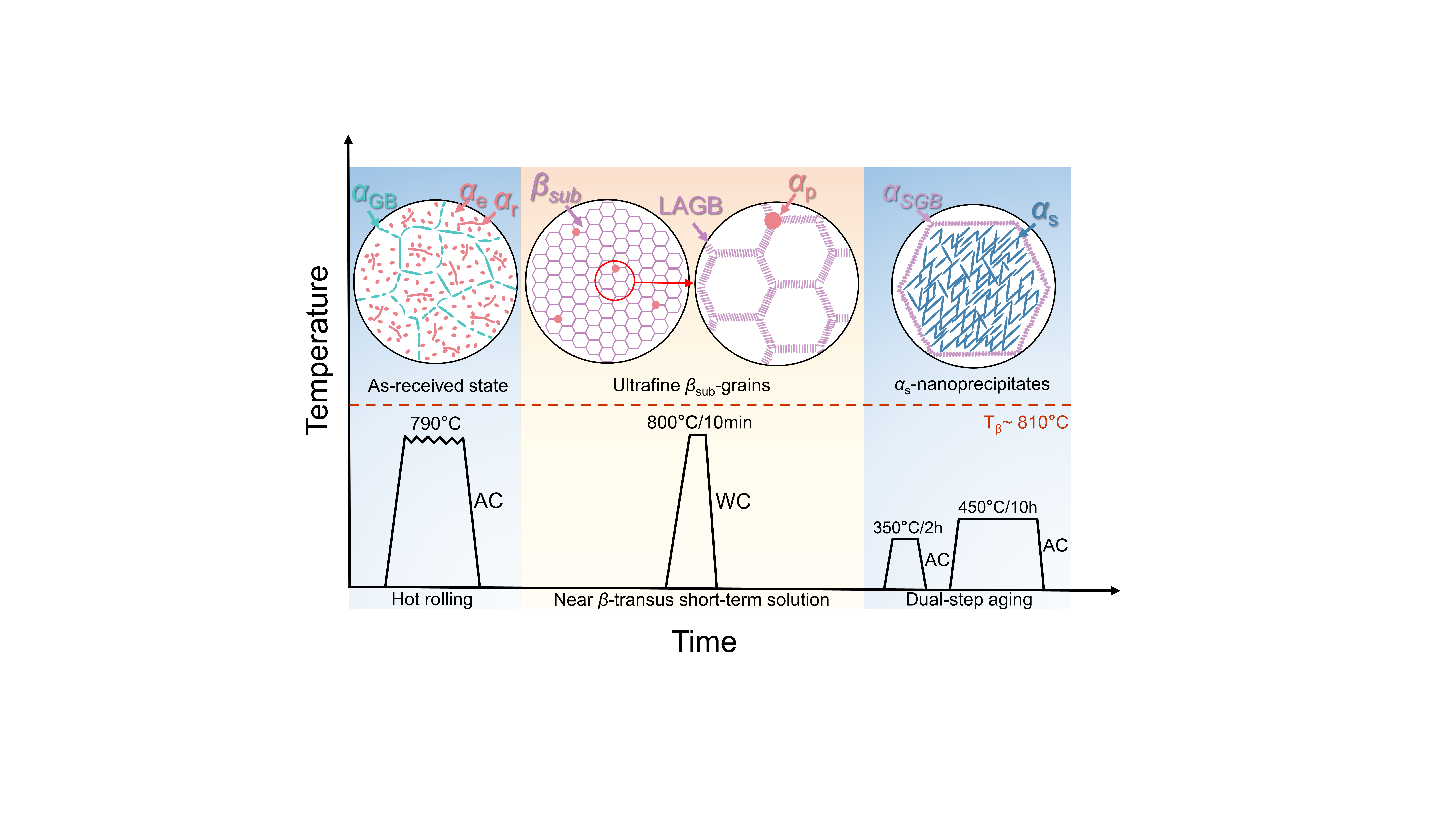


**Figure. S2.** Schematic illustration of the designed near *β*-transus short-term solution plus dual-step aging route to construct a microstructure featuring nanoscale *α*-precipitates (*α_s_*) embedded within ultrafine *β*_sub_-grains (*β*_sub_) in Ti-4Al-5Mo-3V-5Cr-1Fe titanium alloy. The as-received hot-rolled microstructure comprises equiaxed *α* (*α*_e_), rod-shaped *α* (*α*_r_) and grain-boundary *α* (*α*_GB_) in the *β*-matrix, while this microstructure is transformed into one containing numerous ultrafine *β*_sub_-grains decorated by sparse equiaxed primary *α* (*α*_p_) at their boundaries or interiors through near *β*-transus short-term solution. Following dual-step aging treatment, dense *α*_s_-nanoprecipitates and subgrain-boundary *α* (*α*_SGB_) nanolaths appear. Particularly, *α*_SGB_ nanolaths are distributed discontinuously along *β*_sub_-grain boundaries (*β*-SGB). Due to the extremely small fraction of *α*_p_ in Ti alloys, it will be not considered in subsequent microstructural characterizations and deformation.


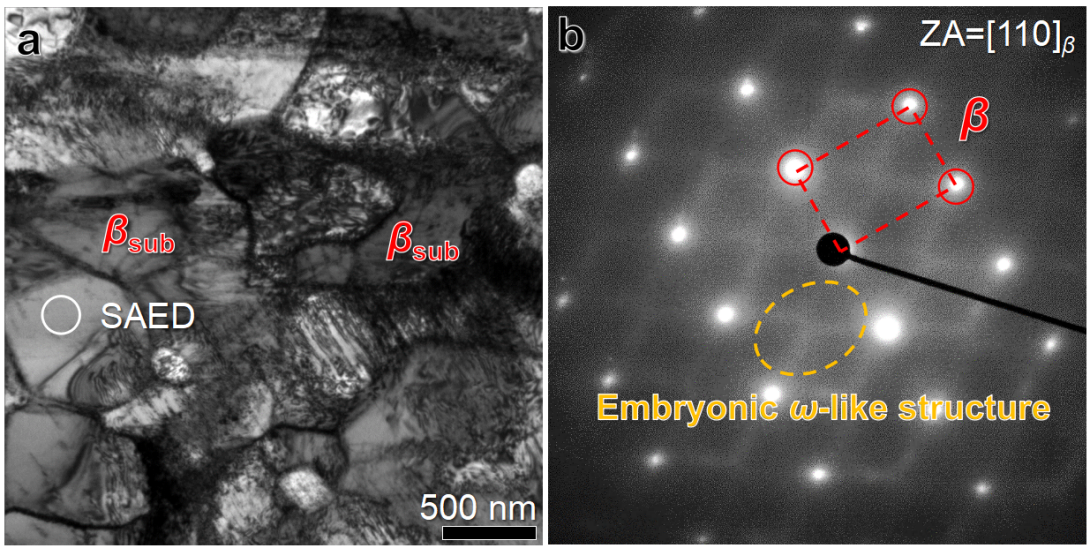


**Figure. S3.** The embryonic *ω*-like structure formed in the NS-STA samples after the near *β*-transus solution treatment (800 ℃/10 min/WC). (a) The TEM bright-filed (BF) image, showing *β_sub_*-grains associated with dense dislocations. The embryonic *ω*-like structure is morphologically indistinct due to its extremely small size. (b) Corresponding SAED pattern taken from the interior of a *β_sub_*-grain along the [110]*_β_* zone axis, showing faint diffuse streaking through the primary *β*-reflections. This diffraction feature is closely related to the formation of embryonic *ω*-like structure from the *β*-matrix upon water quenching.


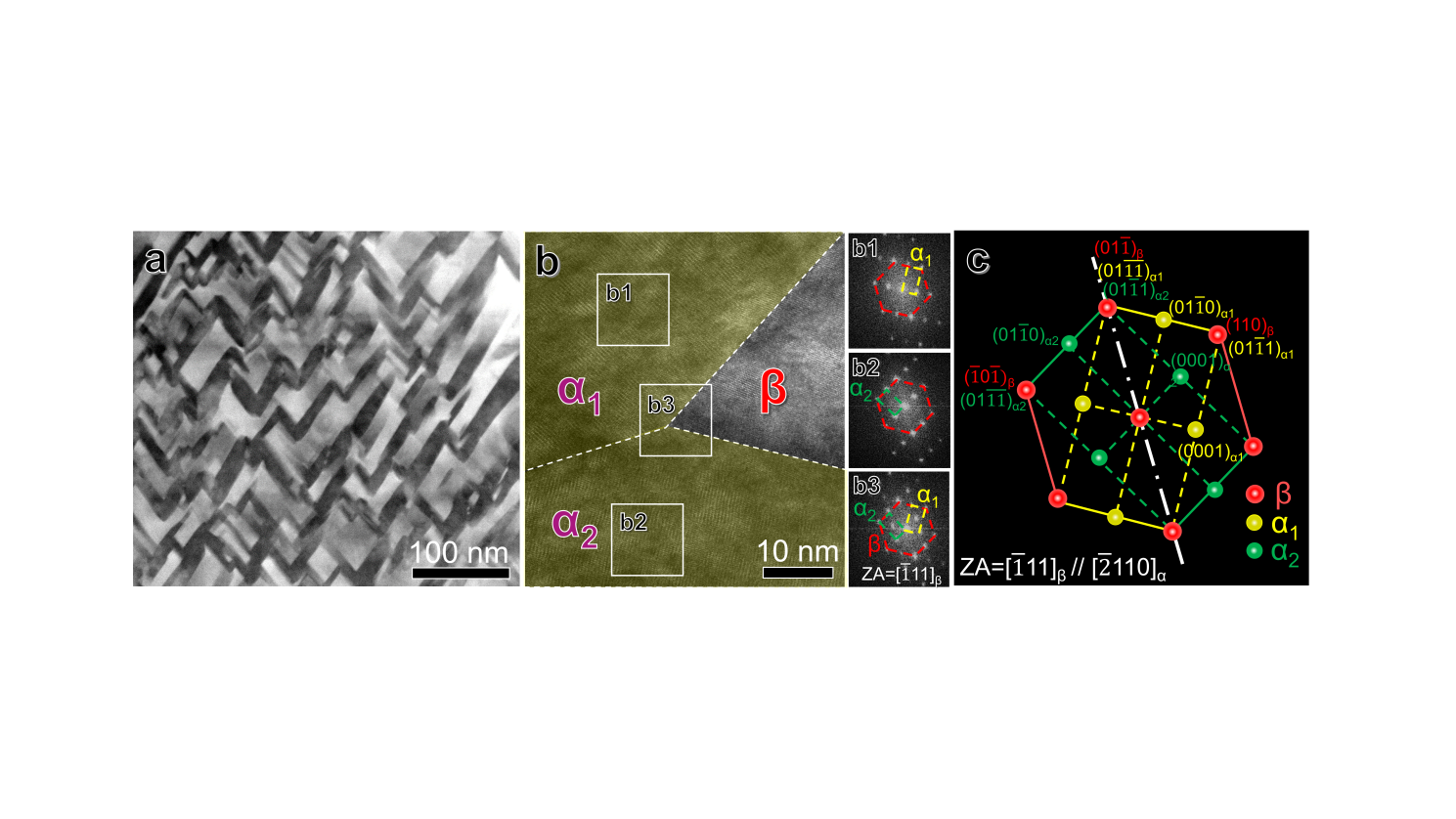


**Figure. S4.** The feature of *α_s_*-nanoprecipitates within *β_sub_*-grain interiors of the NS-STA samples after dual-aging treatment. (a) The HAADF image showing that *α_s_*-nanoprecipitates predominantly exist as chevron-like pairs formed by two *α*-variants. (b) The HRTEM image of *α_s_*-nanoprecipitates displaying a representative chevron-like *α_s_*-pair under the ${[\bar{1}11]}_{\beta}$ zone axis. Panels ‘b1’ and ‘b2’ are FFT images of *α*_1_-variant and *α*_2_-variant, respectively, while panel ‘B3’ is a FFT image taken from the interface between the *α*-pair and *β*-matrix. (c) Key diagram associated with panels ‘b1’, ‘b2’ and ‘b3’. The two *α*-variants in the chevron-like *α_s_*-pair demonstrate a twinning relationship characterized by a twin plane of ${\{1\bar{1}01\}}_{\alpha}$.


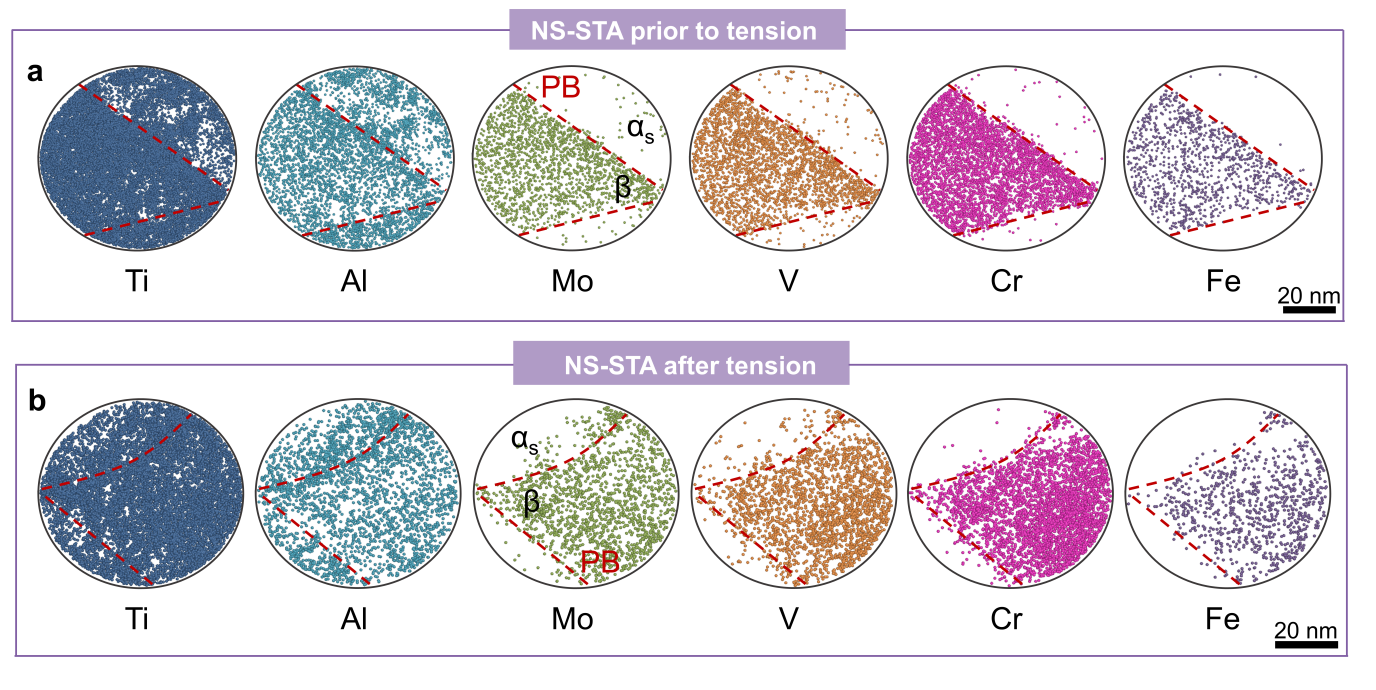


**Figure. S5.** 2D in-plane atomic distribution maps of NS-STA samples obtained from APT results. (a) The *α_s_*/*β* interfaces are relatively clear and sharp prior to tension. (b) The *α_s_*/*β* interfaces become diffusive and ambiguous after tension.


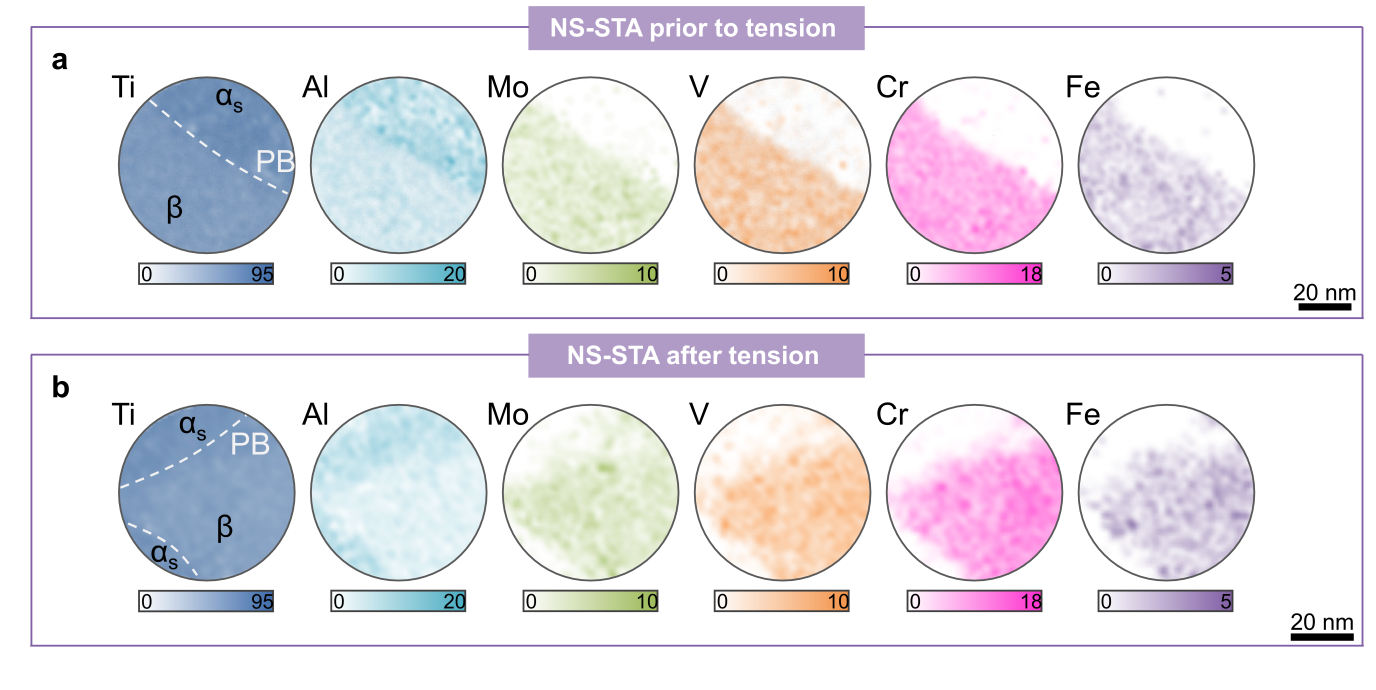


**Figure. S6.** 3D-APT reconstructed atom maps of NS-STA samples showing the composition distribution before and after tension. (a) Reconstructed atom maps prior to tension, revealing the nanoscale local chemical heterogeneities in the *β*-matrix, in particular the Mo, V, Cr and Fe elements. (b) Reconstructed atom maps after tension. Dislocations movement exacerbates the chemical heterogeneities in the *β*-matrix. The fluctuation of Mo, V, Cr and Fe are more obvious.


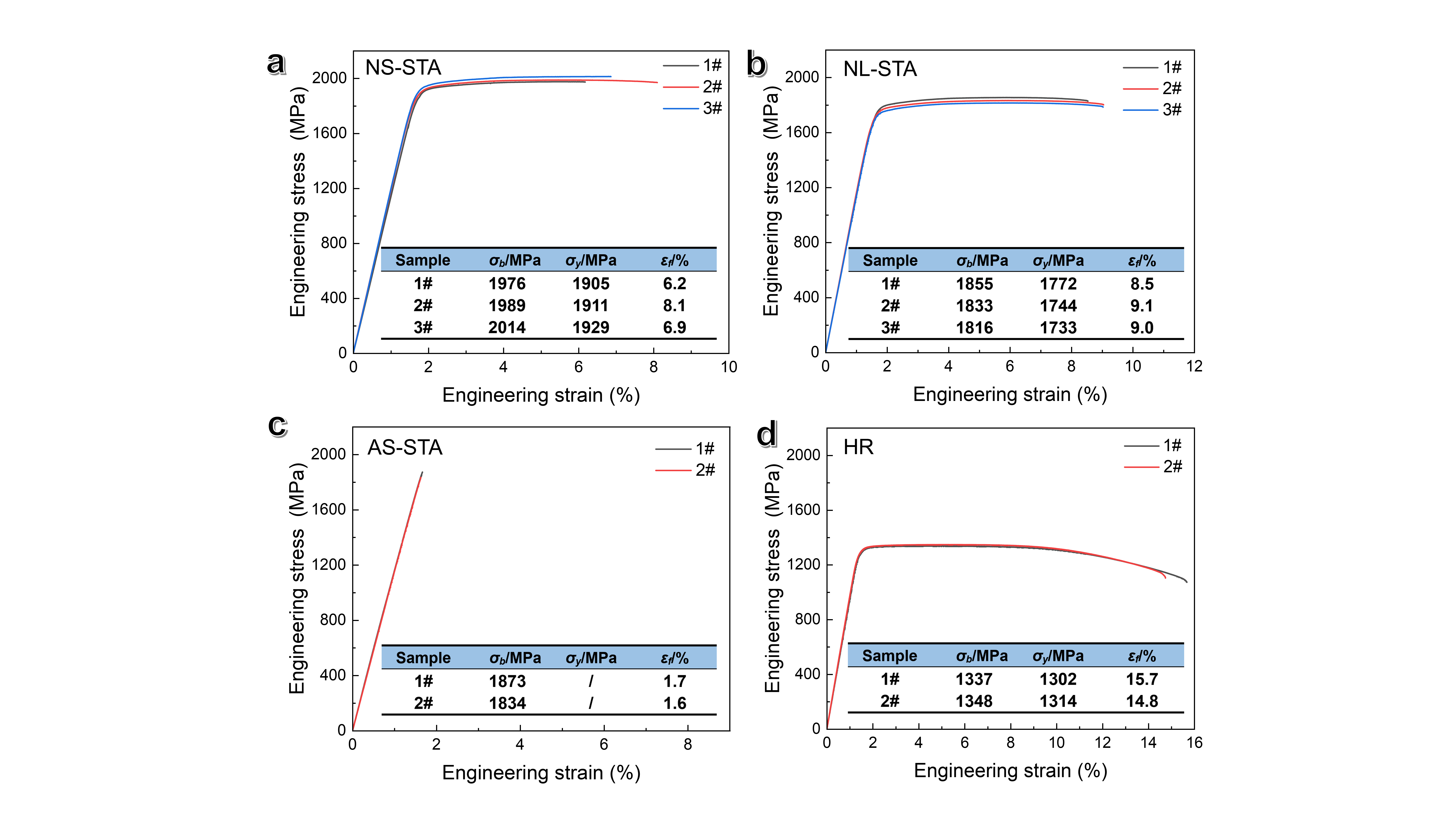


**Figure. S7.** Engineering stress-strain curves of our four types of alloys. (a) The NS-STA samples. (b) The NL-STA samples. (c) The AS-STA samples. (d) The HR samples. Tensile testing was conducted repeatedly at each state, with the corresponding tensile data presented in the insets, demonstrating good repeatability of the samples’ tensile behaviors across all states.


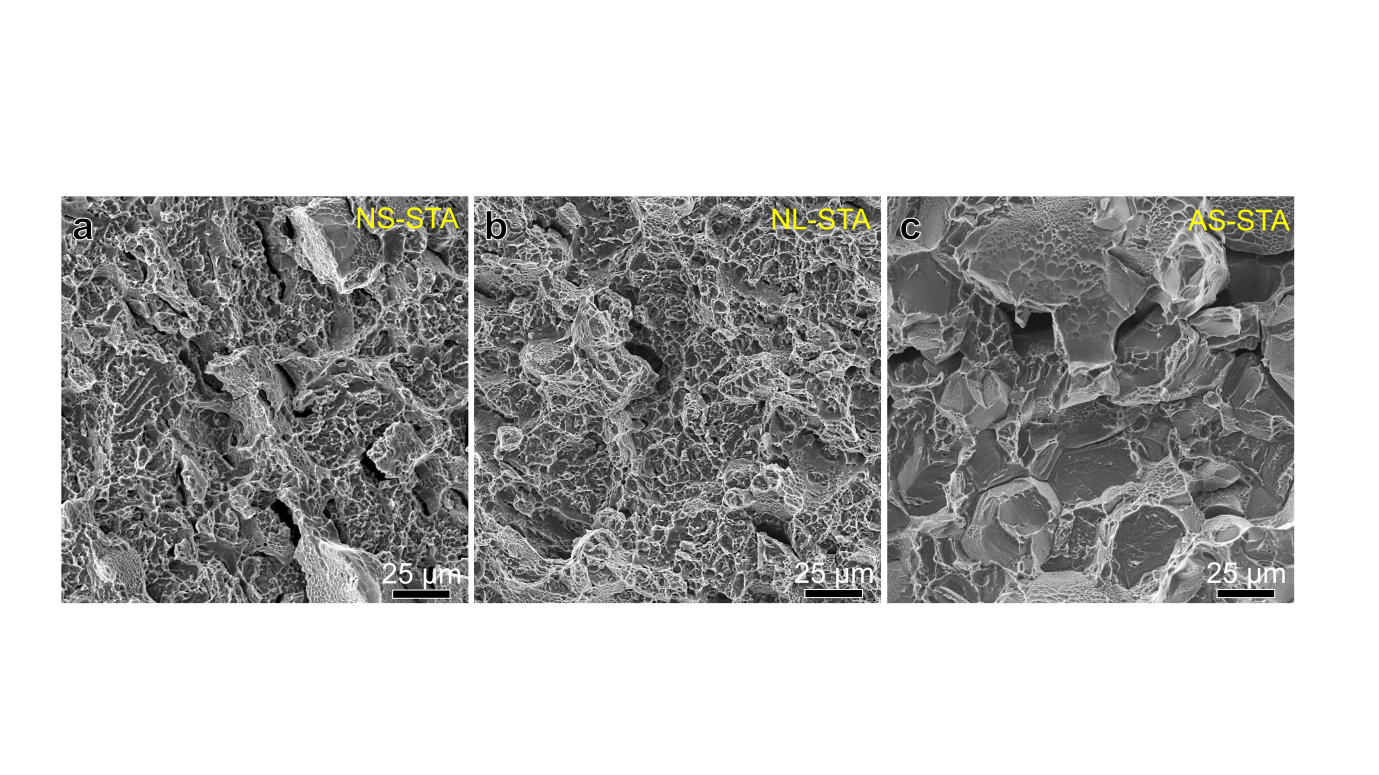


**Figure. S8.** Fractographies of different samples after ambient-temperature tension. (a) The NS-STA samples. (b) The NL-STA samples. (c) The AS-STA samples. Both NS-STA samples and NL-STA samples display intragranular ductile fracture. The fracture surfaces are characterized by numerous dimples, indicating substantial plastic deformation during tensile loading. Compared with the NS-STA samples, the NL-STA samples exhibit denser dimples, which is consistent with their higher tensile ductility. In contrast, the AS-STA samples show intergranular brittle fracture, corresponding to a sudden fracture failure during the elastic deformation stage.


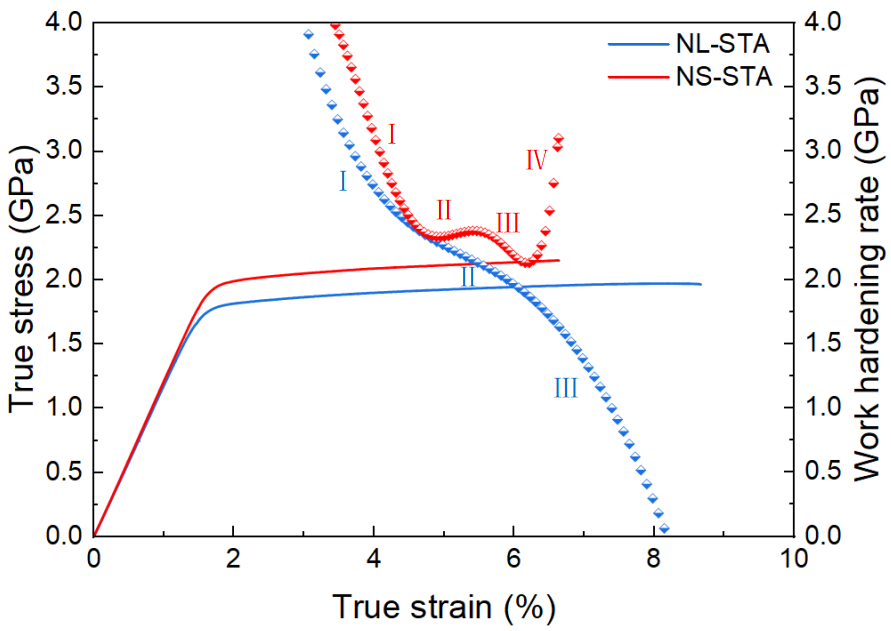


**Figure. S9.** Multi-stage work hardening behaviors for the NL-STA and NS-STA samples. Various work hardening stages are denoted by capital Roman numerals.


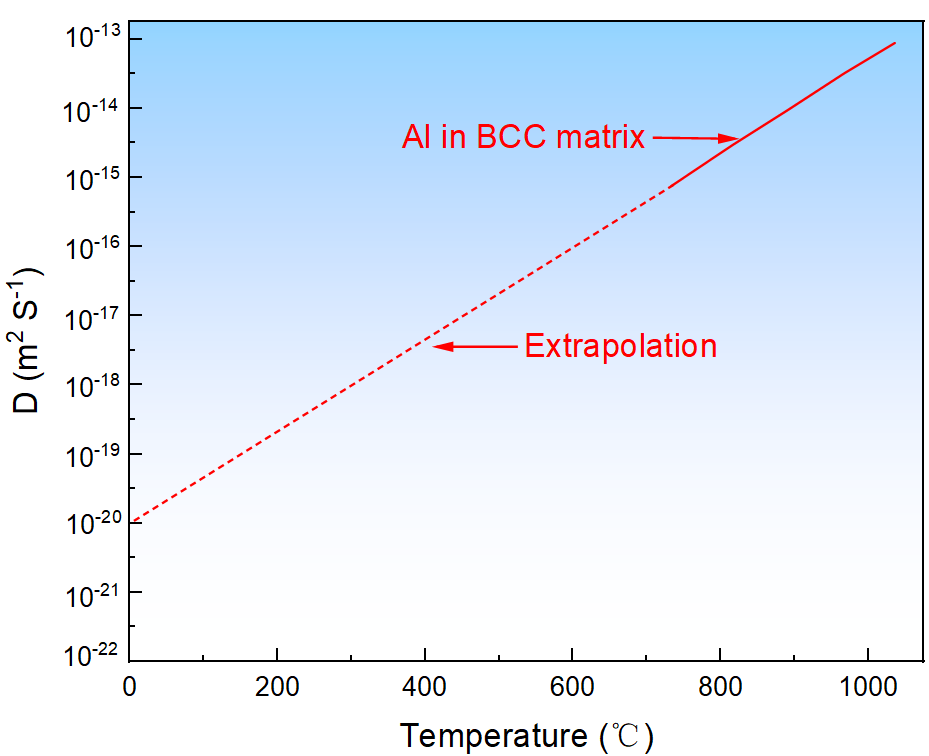


**Figure. S10.** Diffusion coefficient of Al atom as a function of temperature in BCC-Ti. The dotted line represents an extrapolation based on available high-temperature data.


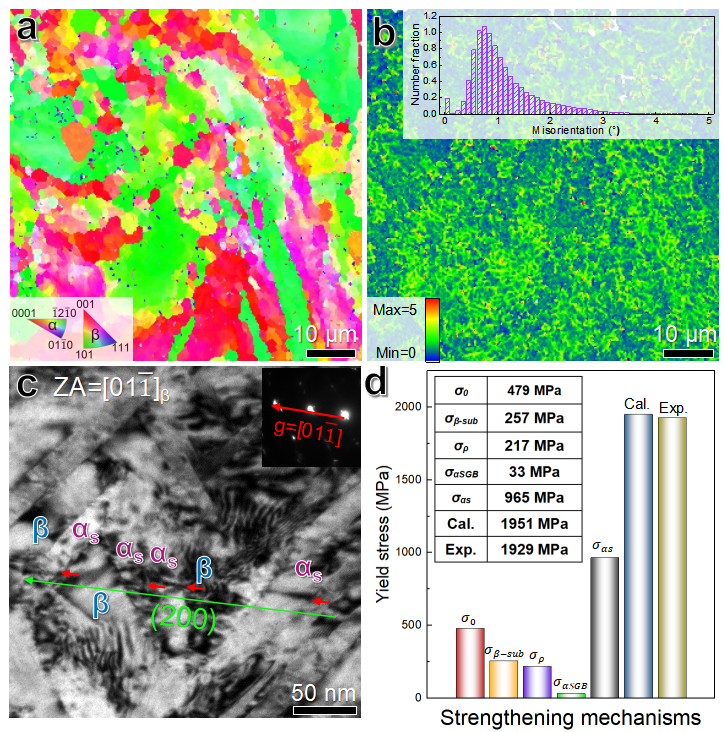


**Figure. S11.** A comparison between the calculated yield strength *σ_y_* and experimentally measured values of the NS-STA samples. (a) and (b) EBSD IPF map and corresponding KAM map of the NS-STA samples prior to tension, providing essential information for the calculation of *σ_ρ_*. The inset is the histogram of local misorientation distribution. (c) A TEM image of dislocation lines under the two-beam condition in the deformed NS-STA samples, offering several information for the calculation of *σ_αs_*. (d) The contributions to yield strength from solid solution strengthening (*σ_0_*), subgrain boundary strengthening (*σ_β-sub_*), Taylor strengthening (*σ_ρ_*), intragranular *α_s_* precipitation strengthening (*σ_αs_*), and intergranular *α_SGB_* precipitation strengthening (σ*_αSGB_*). Histograms comparing the calculated *σ_y_* with the experimentally measured value demonstrate good agreement between them.


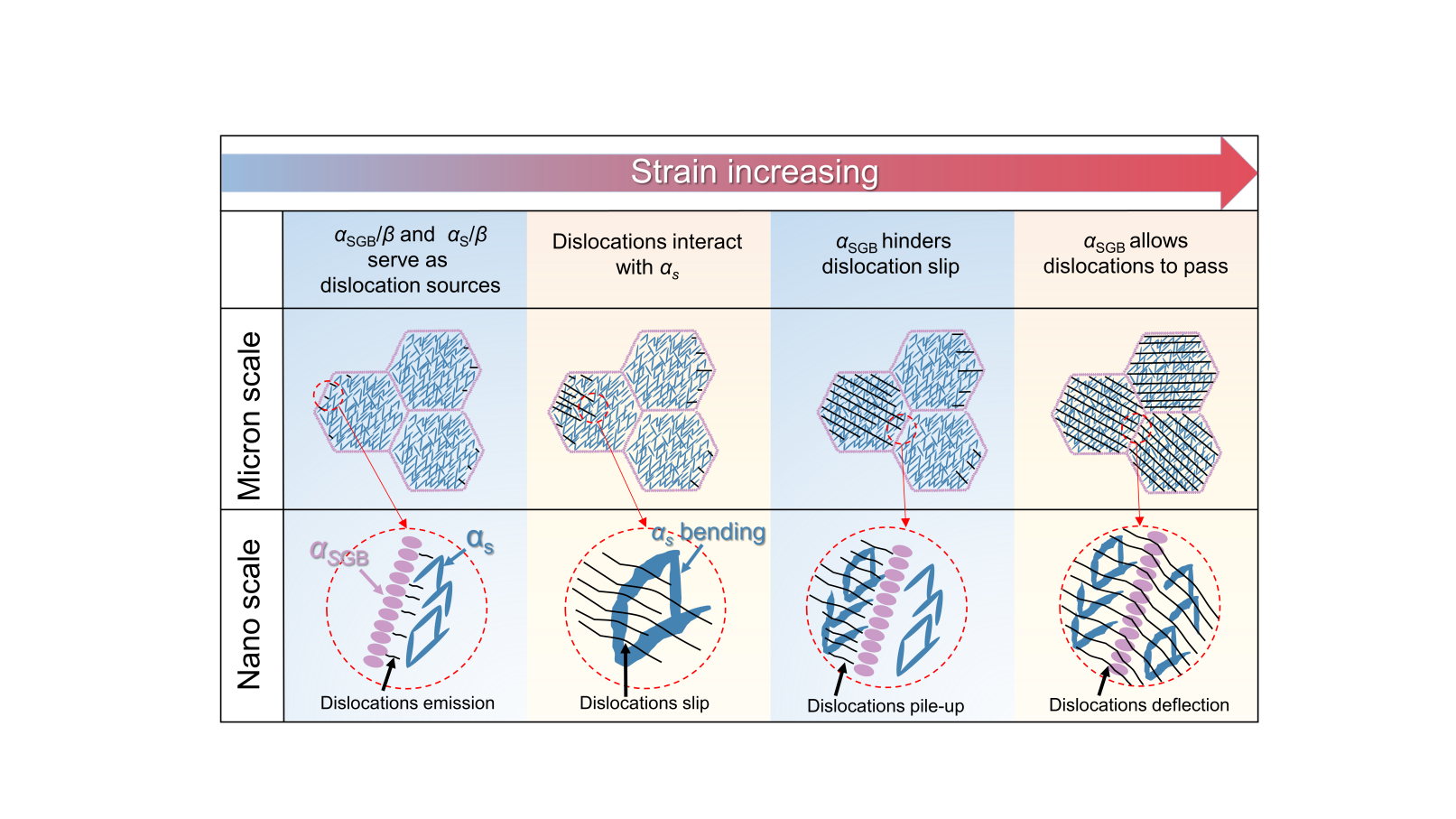


**Figure. S12.** Schematic illustration of the deformation process in the NS-STA samples upon straining. It highlights the roles of both *α_s_*-nanoprecipitates and ultrafine *β_sub_*-grains in accommodating plastic deformation in the alloy material.

**Supplementary Table**

**Table S1.** Physical parameters and mechanical features used for strength calculations of the NS-STA samples.

| **Parameter** | **Physical description** | **Value** | **Ref.** |
| --- | --- | --- | --- |
| *σ_CRSS_* | Shear stress of pure Ti | 180 MPa | ^[15]^ |
| *Bi* | Strengthening coefficient | / | ^[15]^ |
| *L* | Average *β*-subgrains size | 613 nm | This work |
| $\theta_{mean}^{LAGB}$ | Average misorientation angle of LAGBs | 2.73° | This work |
| *M* | Taylor factor | 2.8 | ^[15]^ |
| *α* | Dislocation interaction constant | 0.3 | ^[15]^ |
| *G* | Shear modulus | 39 GPa | ^[15]^ |
| *b* | Magnitude of Burgers vector | 2.8 Å | ^[15]^ |
| *f_HAGB_* | The fraction of HAGBs | 6% | This work |
| *X* | Step size | 0.25 μm | This work |
| *θ_KAM_* | Average KAM values | 1.12° | This work |
| *h* | Average thickness of the *β*-matrix | 16 nm | This work |
| *n* | Number of dislocations crossing the *α_s_*-lamellae | 4 | This work |

**References**

[1] Q. Ding, Y. Zhang, X. Chen, X. Fu, D. Chen, S. Chen, L. Gu, F. Wei, H. Bei, Y. Gao, M. Wen, J. Li, Z. Zhang, T. Zhu, R.O. Ritchie, Q. Yu, Tuning element distribution, structure and properties by composition in high-entropy alloys, *Nature* **2019**, 574, 223.

[2] E. Ma, C. Liu, Chemical inhomogeneities in high-entropy alloys help mitigate the strength-ductility trade-off, *Prog. Mater Sci.* **2024**, 143, 101252.

[3] B.C. Wyatt, Y. Yang, P.P. Michałowski, T. Parker, Y. Morency, F. Urban, G. Kadagishvili, M. Tanwar, S.P. Muhoza, S.K. Nemani, A. Bedford, H. Fang, Z.D. Hood, J. Jang, K. Kamath, B.G. Wright, R. Disko, A. Thakur, S. Han, N. Ghosh, X. Xu, Z. Fakhraai, Y. Gogotsi, A. Vojvodic, D.-e. Jiang, B. Anasori, Order-to-disorder transition due to entropy in layered and 2D carbides, *Science* **2025**, 389, 1054.

[4] Y. Xing, D. Zhao, J. Lei, Y. Mao, Z. Zheng, W. Chen, J. Zhang, X. Liu, J. Sun, Enhancing strength-ductility synergy in metastable β-Ti alloys through β-subgrains-mediated hierarchical α-precipitation, *J. Mater. Sci. Technol.* **2025**, 218, 211.

[5] M. Ahmed, T. Li, G. Casillas, J.M. Cairney, D. Wexler, E.V. Pereloma, The evolution of microstructure and mechanical properties of Ti–5Al–5Mo–5V–2Cr–1Fe during ageing, *J. Alloys Compd.* **2015**, 629, 260.

[6] D. Banerjee, J.C. Williams, Perspectives on Titanium Science and Technology, *Acta Mater.* **2013**, 61, 844.

[7] G. Wu, C. Liu, Y.-Q. Yan, S. Liu, X. Ma, S. Yue, Z.-W. Shan, Elemental partitioning-mediated

rystalline-to-amorphous phase transformation under quasi-static deformation, *Nat. Commun.*

**2024**, 15, 1223.

[8] L. Zhao, N. Park, Y. Tian, A. Shibata, N. Tsuji, Deformation-assisted diffusion for the enhanced kinetics of dynamic phase transformation, *Mater. Res. Lett.* **2018**, 6, 641.

[9] S. Ma, W. Wang, S. Zhang, Z. Zhang, H. Liu, B. Wang, The diffusion characteristics of Fe-Ti during shear-extrusion solid-state bonding and the influence of the level of deformation, *Mater. Des.* **2025**,257,114382.

[10] G. Smirnov, Non-Arrhenius diffusion in bcc titanium: Vacancy-interstitialcy model, *Phys. Rev. B* **2020**, 102, 184110.

[11] G. Stechauner, E. Kozeschnik, Assessment of substitutional self-diffusion along short-circuit paths in Al, Fe and Ni, *Calphad* **2014**, 47, 92.

[12] J. Chen-Min Li, Physical chemistry of some microstructural phenomena, *Metall. Trans. A* **1978**, 9, 1353.

[13] C. Zhang, X. Bao, M. Hao, W. Chen, D. Zhang, D. Wang, J. Zhang, G. Liu, J. Sun, Hierarchical

nano-martensite-engineered a low-cost ultra-strong and ductile titanium alloy, *Nat. Commun.*

**2022**, 13, 5966.

1. H. Zhang, J. Zhang, S. Liu, D. Zhang, G. Liu, J. Sun, Designing ultra-strong and ductile hierarchical titanium alloys via interstitial solute-mediated multi-morphologic α-nanoprecipitates, *Acta Mater.* **2023**, 255, 119082.

[15] G.H. Zhao, X.Z. Liang, B. Kim, P.E.J. Rivera-Díaz-del-Castillo, Modelling strengthening mechanisms in beta-type Ti alloys, *Mater. Sci. Eng., A* **2019**, 756, 156.

[16] P. Luo, D.T. McDonald, W. Xu, S. Palanisamy, M.S. Dargusch, K. Xia, A modified Hall–Petch relationship in ultrafine-grained titanium recycled from chips by equal channel angular pressing, *Scr. Mater.* **2012**, 66, 785.

[17] G.-H. Zhao, X. Xu, D. Dye, P.E.J. Rivera-Díaz-del-Castillo, Microstructural evolution and strain-hardening in TWIP Ti alloys, *Acta Mater.* **2020**, 183, 155.

[18] C. Zhang, S. Liu, J. Zhang, D. Zhang, J. Kuang, X. Bao, G. Liu, J. Sun, Trifunctional nanoprecipitates ductilize and toughen a strong laminated metastable titanium alloy, *Nat. Commun.* **2023**, 14, 1397.

[19] L. Fan, T. Yang, Y. Zhao, J. Luan, G. Zhou, H. Wang, Z. Jiao, C.-T. Liu, Ultrahigh strength and ductility in newly developed materials with coherent nanolamellar architectures, *Nat. Commun.* **2020**, 11, 6240.
